# The apicobasal dispersion of ventricular repolarization in humans is associated with age and affects arrhythmia vulnerability

**DOI:** 10.1113/JP288356

**Published:** 2025-05-28

**Authors:** Vladimír Sobota, Job Stoks, Kiran Haresh Kumar Patel, Roshni Shetty, Haibo Ni, Eleonora Grandi, Fu Siong Ng, Paul G. A. Volders, Matthijs J. M. Cluitmans, Jason D. Bayer

**Affiliations:** ^1^ IHU Liryc, Electrophysiology and Heart Modeling Institute Fondation Bordeaux Université Bordeaux France; ^2^ Institut de Mathématiques de Bordeaux University of Bordeaux Talence France; ^3^ Department of Cardiology, Cardiovascular Research Institute Maastricht (CARIM) Maastricht University Medical Center Maastricht The Netherlands; ^4^ National Heart and Lung Institute (NHLI) Imperial College London London UK; ^5^ Department of Pharmacology University of California Davis Davis CA USA

**Keywords:** computer simulation, electrocardiographic imaging, repolarization gradient, sex‐specific electrophysiology, ventricular fibrillation

## Abstract

**Abstract:**

The apicobasal repolarization gradient (ABRG) plays an important role in determining the sequence of ventricular repolarization, but the effects of sex and age on ABRG are unknown. In this study, we investigate the age‐ and sex‐related differences in ABRG and evaluate their possible role in vulnerability to arrhythmia. Electrocardiographic imaging was performed in 22 healthy subjects (16 females and 6 males) during sinus rhythm, and the average recovery time (RT) at the ventricular apex and base was determined. Fifty‐six different ABRGs were simulated in a male and female model of human ventricular epicardium with sex‐specific electrophysiology by simultaneously adjusting the apicobasal gradients of the slow and rapid delayed rectifier potassium currents. The models were burst paced from the ventricular apex and right ventricular outflow tract to assess the effect of ABRGs on arrhythmia vulnerability. Apicobasal differences in RT (human subjects) and repolarization time (simulation data) were calculated to quantify the ABRGs. In human subjects, ABRGs diminished and eventually inverted (longer RT at the apex than at the base) with increasing age (*r* = −0.7265, *P* = 0.0001). In both male and female models, apical pacing resulted in arrhythmia in 20/ 56 simulations, whereas right ventricular outflow tract pacing resulted in arrhythmia in 15/56 simulations. Arrhythmias were attributable to re‐entry from unidirectional block and generally lasted longer in the models with shorter RT at the apex than at the base. Our findings demonstrate that the ABRG diminishes or inverts with ageing in both male and female human ventricles, which can reduce vulnerability to re‐entrant ventricular arrhythmia.

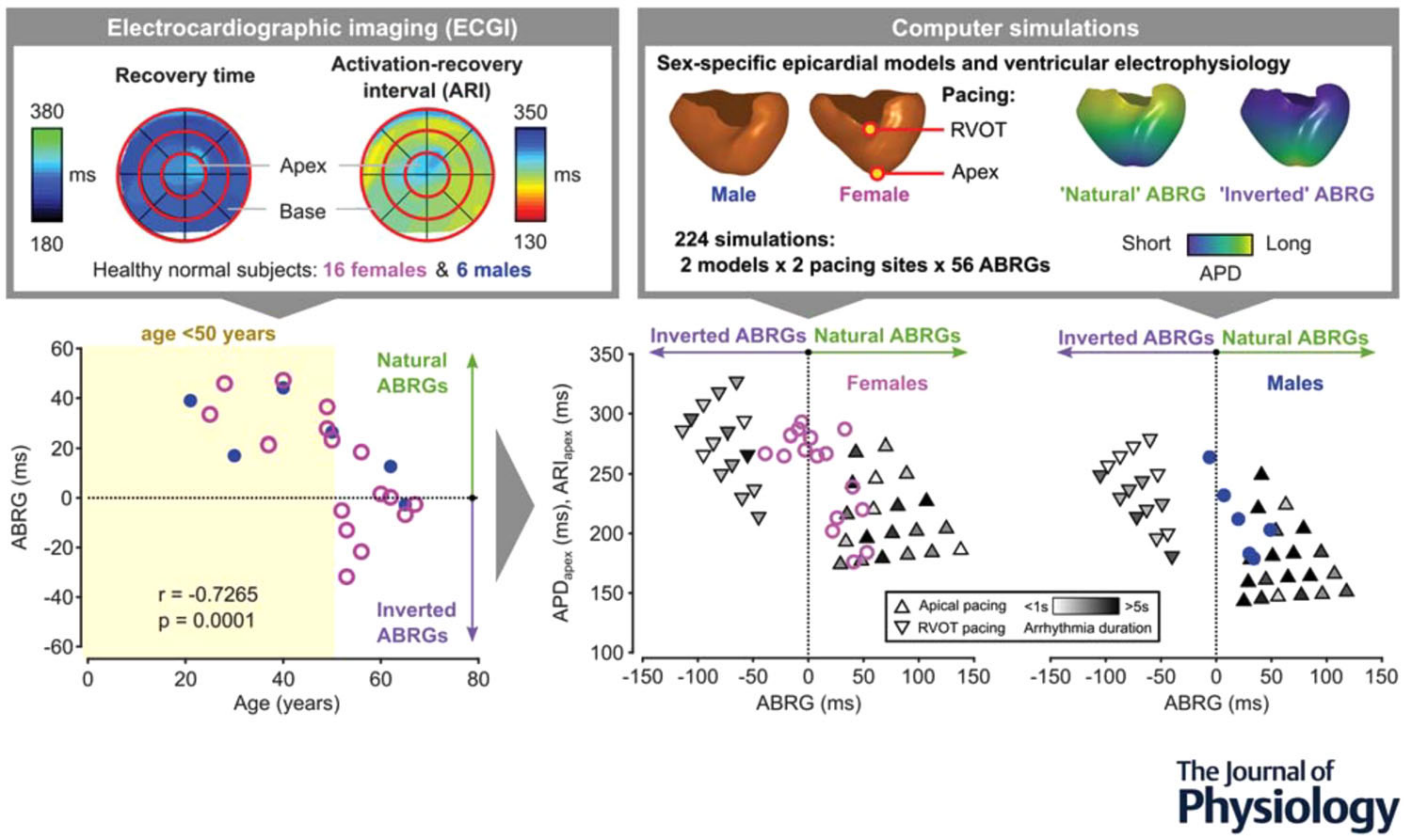

**Key points:**

- The apicobasal repolarization gradient (ABRG) determines the sequence of ventricular repolarization.
- Little is known about ABRG variability in humans and the effects of sex and age on the ABRG.
- Using electrocardiographic imaging data from healthy human subjects, we found that ABRG diminishes or inverts with ageing, in both males and females.
- Our simulations in computational models of human ventricular epicardium show that diminishing and inverting the ABRG is associated with a low vulnerability to arrhythmia.
- By linking together electrocardiographic imaging data and computer simulations, we demonstrate that vulnerability to ventricular arrhythmia might depend on age‐related differences in ABRG.

## Graphical abstract

**Abstract figure legend** We investigated the age‐ and sex‐related differences in the apicobasal repolarization gradient (ABRG) and evaluated their possible role inventricular arrhythmia vulnerability. Electrocardiographic imaging (ECGI) was performed in healthy subjects during sinus rhythm, and the average recovery time (RT) and activation–recovery interval (ARI) at the ventricular apex and base were determined. Different ABRGs were simulated in a male and a female model of human ventricular epicardium. The models were burst paced from the ventricular apex and right ventricular outflow tract (RVOT) to assess the effect of ABRGs on arrhythmia vulnerability. In human subjects, ABRGs diminished and eventually inverted with increasing age. In both male and female models, apical and RVOT pacing resulted in arrhythmia in the presence of steep ABRGs. We demonstrated that the ABRG diminishes or inverts with ageing in both male and female human ventricles, which can reduce vulnerability to re‐entrant ventricular arrhythmia.

## Introduction

Sudden cardiac death due to lethal ventricular arrhythmias represents ∼50% of all cardiovascular deaths in the Western world (Al‐Khatib et al., 2018; Zeppenfeld et al., 2022). Increased or abnormal dispersion of ventricular repolarization has been shown to increase susceptibility to ventricular arrhythmias (Coronel et al., 2009; Rivaud et al., 2021) and has been found in survivors of idiopathic ventricular fibrillation (VF) (Cluitmans et al., 2021). The identification of repolarization heterogeneities that can lead to lethal ventricular arrhythmias is therefore essential for determining patients at risk of and designing preventative measures for sudden cardiac death.

The apicobasal repolarization gradient (ABRG) plays an important role in human ventricular electrophysiology, determining the sequence of ventricular repolarization and T‐wave shape (Ramanathan et al., 2006). The existence of ventricular ABRGs has been shown experimentally in several species, including humans (Janse et al., 2012; Opthof et al., 2016). In a computational study that simulated apicobasal and transmural repolarization gradients (Keller et al., 2012), the ABRG was found to be essential for reproducing a physiologic ECG. At the cellular level, the ABRG is caused by upregulated repolarization currents at the ventricular apex when compared to the base (Szentadrassy et al., 2005).

Our previous work has shown that the presence of a steep ABRG in combination with ventricular hypertrophy can lead to sustained ventricular arrhythmia, irrespective of the presence or absence of diffuse fibrosis (Sobota et al., 2022). These simulations also pointed to a possible interaction of the ABRG with sex‐specific ventricular electrophysiology characterized by longer action potential duration (APD) in females, which lead to sex‐specific differences in vulnerability to ventricular arrhythmia. To date, no study has investigated whether differences in the ABRG exist between males and females.

It is also unknown whether the ABRG changes with ageing. Age‐related changes in ventricular repolarization have been thoroughly described and are characterized by a gradual prolongation of the corrected QT (QTc) interval that is more prevalent in males than in females (Rabkin et al., 2016). These findings suggest age‐related differences in the ABRG, yet no such evidence currently exists.

Recently, Stoks et al. (2024) used electrocardiographic imaging (ECGI) to investigate the variability in ventricular activation and recovery patterns in normal human hearts. They demonstrated that activation and recovery intervals vary profoundly among subjects. In this study, we used the dataset provided by Stoks et al. (2024) to calculate the ABRG for each subject. We then evaluated the relationship between the steepness of the ABRG and age in both males and females. Subsequently, we performed computational simulations in a male and female model of human ventricular epicardium that incorporated sex‐specific ventricular electrophysiology to assess the impact of the ABRG on ventricular arrhythmia vulnerability.

## Methods

### Study population and ECGI measurements

This study is based on previously recorded ECGI data (Stoks et al., 2024). It was approved by the local ethics committees of Maastricht University Medical Centre + (MUMC+), the Netherlands (METC 11‐2‐043, registered at ClinicalTrials.gov, identifier NCT03947021) and the Health Research Authority London (Surrey) Research Ethics Committee, UK (19/LO/0762, registered at ClinicalTrials.gov, identifier NCT03910725). The study adheres to the *Declaration of Helsinki*. Written informed consent was obtained from all subjects prior to inclusion.

ECGI data were used from 22 healthy normal subjects (6 males and 16 females) recorded at MUMC+ and at Imperial College London, UK(Stoks et al., 2024). All subjects were >18 years of age and had a structurally normal heart as verified on the echocardiogram, a normal 12‐lead ECG and no suspected pathology affecting ventricular electrophysiology. In each subject, ∼200 Ag/AgCl electrodes were attached to the participant's chest to record body surface potentials (sampling frequency 2048 Hz) at rest for 10 consecutive sinus beats. Either a non‐contrast low‐dose thoracic CT scan (MUMC+ subjects, *n* = 11) or a 1.5 T MRI scan (Imperial College London subjects, *n* = 11) was performed for each subject to image the heart geometry at the end of diastole, as well as to record the position of the body surface electrodes. Previously validated ECGI methods (Bear et al., 2021; Cluitmans et al., 2017) were used to reconstruct unipolar electrograms on the ventricular epicardial surface. For each unipolar electrogram, the activation time (AT) and recovery time (RT), which is analogous to repolarization time, were determined (Stoks et al., 2023). Activation–recovery intervals (ARIs), a surrogate measure of APD (Stoks et al., 2023), were calculated as ARI = RT − AT.Bullseye plots visualizing ARIs and RTs were generated with the open‐source algorithm UNISYS (Stoks et al., 2020). Average ARIs and RTs for the apical, mid‐myocardial and basal regions were calculated using the data from 10 consecutive sinus beats, and the RT values were plotted for each subject. Additional details regarding the subjects and the ECGI procedure can be found in the respective study (Stoks et al., 2024).

### Computational models of the human ventricular epicardium

**Figure 1:**
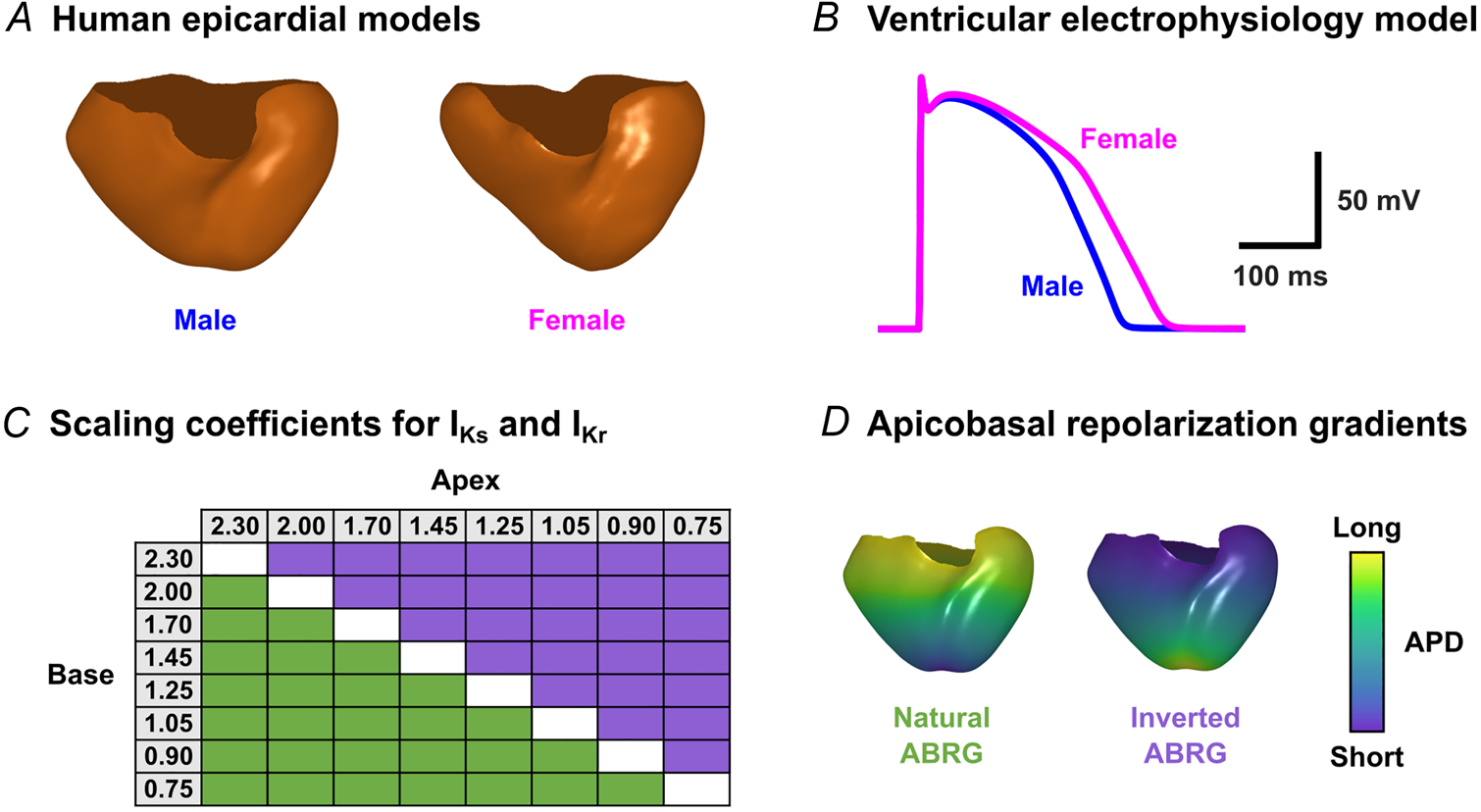
Computational models of the human ventricular epicardium *A*, the male and the female human epicardial meshes used in this study. *B*, epicardial action potentials generated by the cellular model of human ventricular electrophysiology. *C*, combinations of scaling coefficients simultaneously applied to the slow and rapid delayed rectifier potassium currents (*I*_Ks_ and *I*_Kr_, respectively) to model apicobasal repolarization gradients (ABRGs). The combinations labelled in green resulted in natural ABRGs, whereas the combinations labelled in purple resulted in inverted ABRGs. *D*, ABRG examples shown on the male mesh of human ventricular epicardium.

Male and female computational models of the human ventricular epicardium were used to simulate the effect of different ABRG levels on arrhythmia vulnerability (Fig. 1*A*). These models were derived from MRI data of a male and female subject, in line with previously published studies (Moreno et al., 2011; Rivaud et al., 2021). The male model contains 216 910 nodes and 432 926 triangular elements with an average edge length of 383 ± 89 µm. The female model contains 180 060 nodes and 359 269 triangular elements with an average edge length of 393 ± 93 µm. Myocardial fibres (Bayer et al., 2012) and sex‐specific ventricular electrophysiology (Fig. 1*B*; O'Hara et al., 2011; Peirlinck et al., 2021) were incorporated into both models. Tissue conductivity was set according to a previous study (Bayer et al., 2016), resulting in conduction velocities of 89 cm/s along the myocardial fibres and 47 cm/s perpendicular to myocardial fibres at the pacing cycle length of 750 ms.

Spatial differences in apicobasal repolarization, due to the presence of ABRGs occurring from heterogeneity in ion channel expression (Ramanathan et al., 2006), were modelled by applying the same scaling factor to the slow and rapid delayed rectifier potassium currents (*I*_Ks_ and *I*_Kr_, respectively) in the apicobasal direction. We used a similar approach in a previous study to obtain a variety of ventricular repolarization gradients induced by drugs (Rivaud et al., 2021). Fifty‐six different combinations of apical and basal scaling coefficients in the range between 0.75 and 2.30 were used, as described in Fig. 1*C*.

To assess arrhythmia vulnerability, each model was paced from the ventricular apex or at the right ventricular outflow tract (RVOT) with the following protocol. A steady‐state simulation of 1000 ms was proceeded by pre‐pacing (S1) for 10 beats with a basic cycle length of 750 ms. Average APD at the ventricular apex (APD_apex_) and at the RVOT (APD_RVOT_), in addition to average repolarization times, were obtained from the last S1 beat. The model was then paced with a cycle length of 400 ms for 10 beats (S2), followed by S3 trains of 10 pulses with shorter cycle lengths (<300 ms) to attempt arrhythmia induction. If arrhythmia was induced, a monitoring period of 5 s followed the S3 train of pulses to assess arrhythmia sustainability. Arrhythmia was considered inducible when at least one of the S3 trains resulted in re‐entrant activity that lasted >1 s (Arevalo et al., 2016). If more than one S3 train induced arrhythmia, the duration of the longest episode was considered.

All simulations were performed as monodomain using the cardiac electrophysiology simulator openCARP (https://opencarp.org; Plank et al., 2021) running on a DELL workstation (32 cores, Intel Xeon Gold 6226R CPU @ 2.90 GHz, 256 GB of memory) or the Joliot‐Curie TGCC supercomputer. Simulation results were visualized with the software Meshalyzer (https://git.opencarp.org/openCARP/meshalyzer). In total, 224 simulations were performed (two sex‐specific meshes, two pacing sites and 56 different ABRGs), requiring ∼14,100 core hours.

### ABRG calculation

The ABRG was derived from the apicobasal difference in repolarization times. For clarity, we will refer to the repolarization (recovery) times derived from ECGI data as RT_ECGI_ and from the simulation‐derived data as RT_sim_. Using the ECGI data, the ABRG was calculated as the difference between RT_ECGI_ at the ventricular base (RT_ECGI,base_) and the apex (RT_ECGI,apex_), i.e. ABRG_ECGI_ = RT_ECGI,base_ − RT_ECGI,apex_. For the simulation‐derived data, the ABRG was calculated as the difference between RT_sim_ at the RVOT region (RT_sim,RVOT_) and the apex (RT_sim,apex_), i.e. ABRG_sim_ = RT_sim,RVOT_ − RT_sim,apex_. In the remaining text, ABRG without a subscript refers to the ABRG as a general phenomenon, while ABRG_ECGI_ and ABRG_sim_ refer to the ABRGs obtained from the ECGI and simulation data, respectively. For simplicity, we refer to the ABRG_ECGI_ and ABRG_sim_ with longer RT_ECGI_ and RT_sim_ at the base compared with the apex (RT_ECGI,apex_ < RT_ECGI,base_ and RT_sim,apex_ < RT_sim,RVOT_) as ‘natural’, because this ABRG orientation has frequently been reported in literature for ‘normal’ hearts (Keller et al., 2012; Opthof et al., 2016). The ABRG_ECGI_ and ABRG_sim_ showing longer RT_ECGI_ and RT_sim_ at the apex than at the base (RT_ECGI,apex_ > RT_ECGI,base_ and RT_sim,apex_ > RT_sim,RVOT_) will be referred to as ‘inverted’ (Fig. 1*D*). In addition to the repolarization times, we report average ARIs at the ventricular apex (ARI_apex_) and ventricular base (ARI_base_) to allow a comparison with local APD differences (APD_apex_ and APD_RVOT_) obtained from the simulation data.

### Statistics

Spearman's correlation coefficient was used to assess the association between variables. Arrhythmia durations were assessed by the Wilcoxon rank‐sum test. A value of *P* < 0.05 was considered statistically significant. Data are presented as the median and interquartile range.

## Results

Data from 22 subjects (16 females and 6 males) were used. More details about the cohort, including AT and RT maps and ECG examples for each subject, are provided in the original ECGI study (Stoks et al., 2024).

### Apicobasal repolarization gradients determined from ECGI

**Figure 2:**
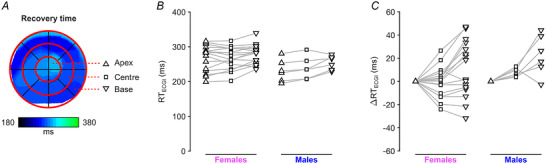
Apicobasal repolarization gradients determined from electrocardiographic imaging data *A*, local recovery times (RT_ECGI_) were obtained from the electrocardiographic imaging (ECGI) data, and average RT_ECGI_ was determined for the apical (RT_ECGI,apex_), central and basal regions. *B*, average RT_ECGI_ for the apical, central and basal regions, plotted independently for female and male subjects. *C*, differences in average RT_ECGI_ with respect to RT_ECGI,apex_. The bullseye plot example presented in *A* was adopted from the original ECGI study (Stoks et al., 2024).

The average RT_ECGI_ at the apex, centre and the base of the ventricles (Fig. 2*A*) was obtained for each subject and plotted separately for males and females (Fig. 2*B*). A large variability in the data was observed, with a general trend towards RT_ECGI,apex_ < RT_ECGI,base_, in both males (5/6) and females (9/16). Normalization of the data with respect to RT_ECGI,apex_ revealed a substantial variability in ABRG_ECGI_, especially in the female subjects, with the differences between RT_ECGI,base_ and RT_ECGI,apex_ spanning from −32 to +47 ms (Fig. 2*C*). The variability in the male subjects was smaller (from −3 to 44 ms), with RT_ECGI,base_ generally being longer than RT_ECGI,apex_. In the subjects ≤50 years of age, median ABRG_ECGI_ was 33 [26–41] ms in females (*n* = 7) and 33 [24–40] ms in males (*n* = 4). In the subjects >50 years of age, the median ABRG_ECGI_ in females (*n* = 9) was −5 [−13 to 0] ms, and the ABRG_ECGI_ in males *(n* = 2) was  −3 and 13 ms. The median apicobasal difference in ARIs (ARI_base_ − ARI_apex_) was 40 [30–45] ms in females (*n* = 7) and 32 [27–38] ms in males (*n* = 4) for the subjects ≤50 years of age, and −6 [−16 to 2] ms (*n*in females >50 years of age (*n* = 9). The apicobasal difference in ARIs was ‐6 and 7 ms in males >50 years of age (*n* = 2).

**Figure 3:**
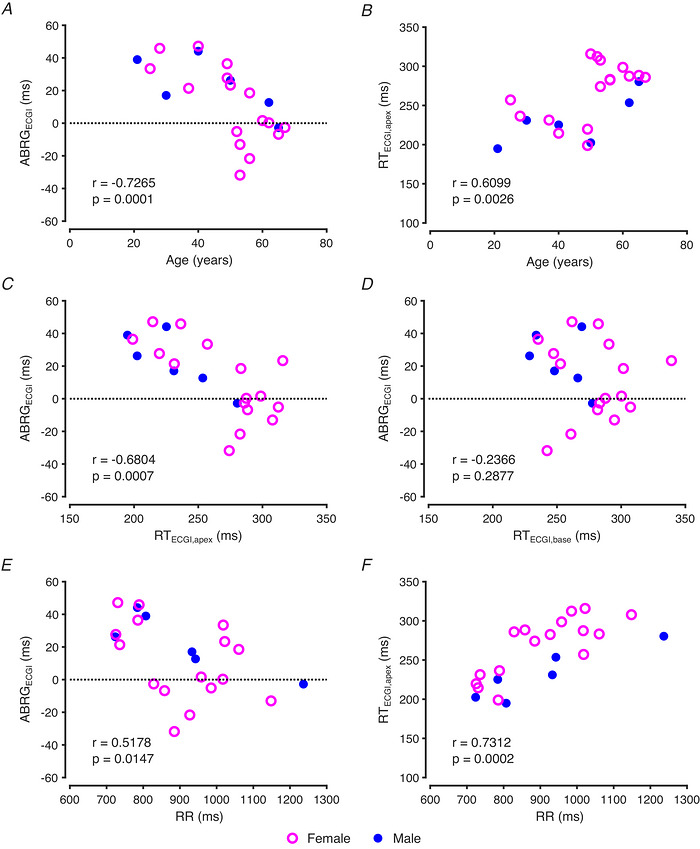
Apicobasal repolarization gradient determined from electrocardiographic imaging data diminishes with age due to prolongation of recovery times at the ventricular apex *A*, a negative association was found between the apicobasal repolarization gradient determined from electrocardiographic imaging data (ABRG_ECGI_) and age. *B*, a positive association was found between average recovery time at the ventricular apex (RT_ECGI,apex_) and age. *C*, ABRG_ECGI_ was negatively correlated with RT_ECGI,apex_. *D*, no association was found between ABRG_ECGI_ and average recovery time at the base (RT_ECGI,base_). *E*, ABRG_ECGI_ was negatively associated with RR interval, indicating that steeper ABRG generally occurred at faster heart rates. *F*, RT_ECGI,apex_ was positively correlated with RR interval, and the same behaviour was found for RT_ECGI,base_ (graph not shown).

A negative association between the ABRG_ECGI_ and age was found (*r* = −0.7265, *P* = 0.0001, Fig. 3*A*), suggesting that ABRG diminishes with ageing and can become inverted in subjects >50 years of age. To investigate the contribution of RT_ECGI,apex_ and RT_ECGI,base_ to this phenomenon, Spearman's correlations between these variables and ABRG_ECGI_ were calculated, revealing a negative association between RT_ECGI,apex_ and ABRG_ECGI_ (*r* = −0.6804, *P* = 0.0007, Fig. 3*C*) and no association between RT_ECGI,base_ and ABRG_ECGI_ (*r* = −0.2366, *P* = 0.2877, Fig. 3*D*). A positive correlation between RT_ECGI,apex_ and age (*r* = 0.6099, *P* = 0.0026, Fig. 3*B*) was found, but there was no association between RT_ECGI,base_ and age (*r* = 0.3355, *P* = 0.1269). These findings demonstrated that the age‐related differences in ABRG_ECGI_ were driven primarily by RT_ECGI,apex_ prolongation.

A tendency for less steep ABRG in subjects with slower heart rate was found, shown by the negative association between the ABRG_ECGI_ and RR interval (*r* = −0.5178, *P* = 0.0147, Fig. 3*E*). Calculating the correlations between the RR interval, RT_ECGI,apex_ and RT_ECGI,base_ showed a positive association of RR with RT_ECGI_ from both regions (*r* = 0.7312, *P* = 0.0002, Fig. 3*F*; and *r* = 0.7064, *P* = 0.0003, respectively), excluding possible region‐specific heart rate‐dependent effects on RT_ECGI_. A summary of all correlations performed between the variables can be found in Table 1.

**Table 1:** Overview of parameter correlations.

Parameter	Age	RR	ABRG_ECGI_	RT_ECGI,apex_
RR	*r* = 0.4047 *P* = 0.0599	—	—	—
ABRG_ECGI_	*r* = −0.7265^a^ *P* = 0.0001	*r* = −0.5178^a^ *P* = 0.0147	—	—
RT_ECGI,apex_	*r* = 0.6099^a^ *P* = 0.0026	*r* = 0.7312^a^ *P* = 0.0002	*r* = −0.6804^a^ *P* = 0.0007	—
RT_ECGI,base_	*r* = 0.3355 *P* = 0.1269	*r* = 0.7064 *P* = 0.0003	*r* = −0.2366^a^ *P* = 0.2887	*r* = 0.8442 *P* < 0.0001

Abbreviations: ABRG_ECGI_, apicobasal repolarization gradient derived from electrocardiographic imaging (ECGI) data; RT_ECGI,apex_, average recovery time at the apex; RT_ECGI,base_, average recovery time at the base.

^a^ Graph shown in Fig. 3.

### Arrhythmia inducibility in the computational models

Fifty‐six different ABRGs were simulated in both the male and female computational models of the human ventricular epicardium. In both the male and the female models, rapid apical pacing resulted in arrhythmia in 20 of 56 simulations, and RVOT stimulation led to arrhythmia in 15 of 56 simulations. Arrhythmias were inducible only by rapid pacing at the regions with the shortest APD. Apical stimulation resulted in arrhythmia only in the models with a natural ABRG, i.e. in models where APD_apex_ was set shorter than APD_base_.Likewise, RVOT pacing resulted in arrhythmia only in the models with an inverted gradient, i.e. where APD_base_ was set shorter than APD_apex_.

**Figure 4:**
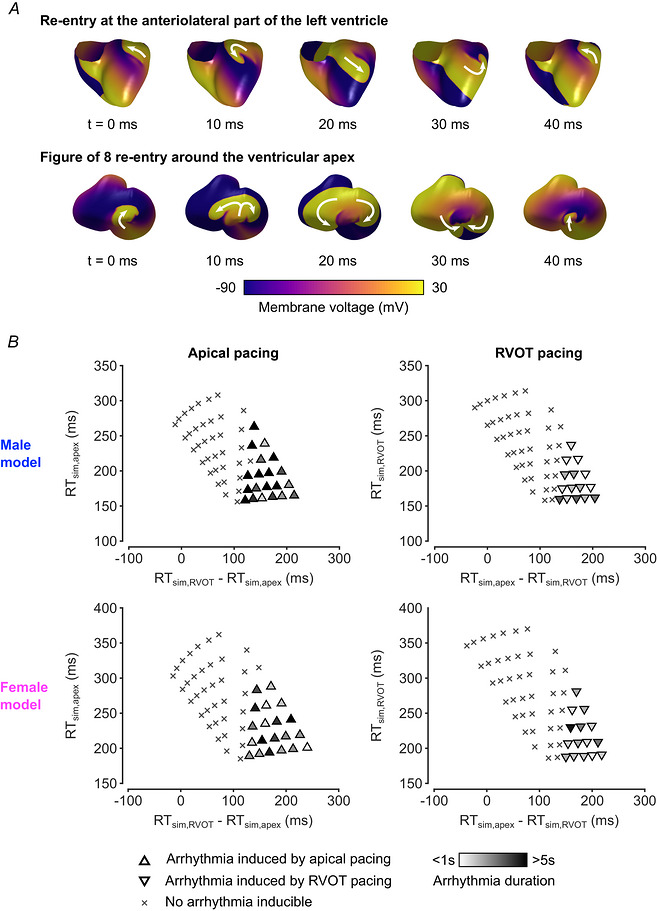
Arrhythmia inducibility in the computational models *A*, examples of re‐entrant arrhythmias observed in the models. *B*, Arrhythmia inducibility during apical and right ventricular outflow tract (RVOT) pacing in the male and female models.

The arrhythmias in the presence of a natural ABRG were attributable to re‐entry localized at the basal portion of the model, such as the left ventricular anterolateral wall (Fig. 4*A*), posterior/posterolateral part of the right ventricle or the region around the RVOT. The arrhythmic episodes <5 s were characterized by multiple simultaneously occurring re‐entry circuits (male model, 2 [1–3] re‐entry circuits, 10/20 episodes; female model, 2 [1–2] re‐entry circuits, 16/20 episodes), and their number gradually decreased in time until the arrhythmia terminated. The episodes that lasted >5 s were also characterized by multiple simultaneous re‐entrant circuits (male model, 3 [2–3] re‐entry circuits, 10/20 episodes; female model, 1 and 2 re‐entry circuits, 2/20 episodes), but they gradually degraded to one sustained re‐entrant circuit that drove the arrhythmia. In the female model, 2 of the 20 arrhythmic episodes in the presence of a natural ABRG were non‐sustained re‐entry. In the presence of an inverted ABRG, arrhythmic episodes were sustained figure‐of‐eight re‐entry around the ventricular apex (male model, 12/15 episodes; female model, 13/15 episodes; Fig. 4*A*). In both male and female models, 2 of 15 arrhythmic episodes in the presence of an inverted ABRG were non‐sustained re‐entry, and one episode in the male model was a single sustained re‐entrant circuit.

**Figure 5:**
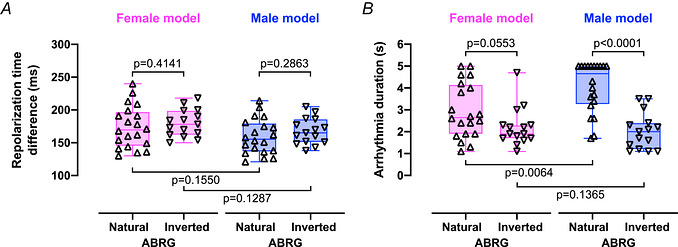
Repolarization times and arrhythmia durations in the male and the female computational models *A*, similar differences in repolarization times (RT_sim_) between the apex (RT_sim,apex_) and base (RT_sim,RVOT_) were observed among the male and the female models whenarrhythmias occurred. Please note that for the natural apicobasal repolarization gradients (ABRG), the RT_sim_ difference was calculated as RT_sim,RVOT_ − RT_sim,apex_, while for the inverted ABRGs it was calculated as RT_sim,apex_ − RT_sim,RVOT_. *B*, overall, longer arrhythmia episodes occurred in the presence of natural ABRGs compared to inverted ABRGs. The arrhythmias in the presence of natural ABRGs lasted longer in the male model than in the female model.

Arrhythmias were inducible only within a critical range of repolarization times at the stimulation site (RT_sim,apex_ or RT_sim,RVOT_) and the difference between RT_sim,apex_ and RT_sim,RVOT_, as shown in Fig. 4*B*. In the male model, rapid apical pacing in combination with a natural ABRG led to arrhythmias only when the difference between RT_sim,RVOT_ and RT_sim,apex_ exceeded 121 ms. The shortest difference between RT_sim,apex_ and RT_sim,RVOT_ in the models with an inverted ABRG where arrhythmias were induced by RVOT pacing was 138 ms. In the female model, apical pacing led to arrhythmias in the presence of natural ABRGs when the difference between RT_sim,RVOT_ and RT_sim,apex_ was >130 ms. RVOT pacing in the models with inverted ABRGs resulted in arrhythmias only when the difference between RT_sim,apex_ and RT_sim,RVOT_ exceeded 150 ms. Overall, in the models for which an arrhythmia occurred, the magnitude of the differences between RT_sim,apex_ and RT_sim,RVOT_ tended to be smaller in the presence of a natural than an inverted ABRG (male models, 156 [137–180] *vs*. 165 [151–186], *P* = 0.2863; female models, 170 [145–197] *vs*. 178 [162–199], *P* = 0.4141). However, the statistical comparisons did not show significant differences between the gradients (Fig. 5*A*).

Arrhythmia duration was longer in the presence of natural ABRGs than inverted ABRGs (Fig. 5*B*). This difference was observed in the male model (4.7 [3.3–5.0] *vs*. 2.0 [1.2–2.4] s, *P* < 0.0001) and was borderline insignificant in the female model (2.7 [1.9–4.2] *vs*. 1.9 [1.7–2.3] s, *P* = 0.0553). Arrhythmias in the presence of natural ABRGs lasted longer in the male model than in the female model (4.7 [3.3–5.0] *vs*. 2.7 [1.9–4.2] s, *P* = 0.0064), indicating a possible sex‐specific difference in arrhythmia vulnerability related to the presence of a natural ABRG. The arrhythmia durations in the presence of inverted ABRGs were comparable between the male and female models (1.8 [1.1–2.3] *vs*. 2.2 [1.7–3.0] s, *P* = 0.1365).

### ABRGs in normal subjects and a predicated increase in arrhythmia vulnerability

**Figure 6:**
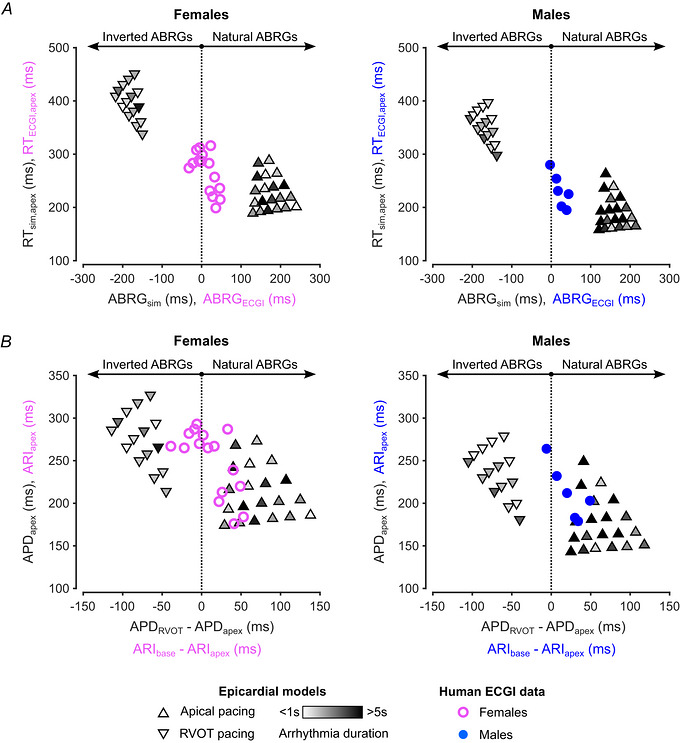
Computer simulations in which an arrhythmia was inducible superimposed on the electrocardiographic imaging data from normal human subjects *A*, apicobasal repolarization gradients (ABRGs) obtained from electrocardiographic imaging (ABRG_ECGI_) and computer simulations (ABRG_sim_) plotted against the respective repolarization times at the apex (RT_ECGI,apex_ and RT_sim,apex_). For both males and females, ECGI and simulation data are located in non‐overlapping clusters. *B*, apicobasal differences in the activation–recovery interval (ARI_base _− ARI_apex_) and action potential duration (APD_RVOT_ − APD_apex_) plotted against the respective ARI or APD at the apex (ARI_apex_ and APD_apex_). Six ECGI data points representing female subjects were found in close proximity to the data points from simulations with natural ABRGs. In the presence of inverted ABRGs, one ECGI data point was found in the proximity of a simulation data point with an arrhythmiaThree ECGI data points representing male subjects were found in close proximity to the simulation data in the presence of natural ABRGs.

To identify ABRGs in normal human subjectsthat are possibly associated with increased arrhythmia vulnerability, the ECGI data were plotted together with the data from computer simulations resulting in arrhythmia. For repolarization times (Fig. 6*A*), the ECGI and simulation data were located in non‐overlapping clusters. However, when apicobasal differences in ARIs and APDs were plotted together against ARI_apex_ and APD_apex_, respectively (Fig. 6*B*), data points from six female and three male subjects were found to be in close proximity of the data points from computer simulations with arrhythmias occuring in the presence of natural ABRGs. The difference between ARI_base_ and ARI_apex_ was found to be ≥22 ms in these subjects (females: 22, 26, 40, 41, 49 and 53 ms; males: 30, 34 and 49 ms). In addition, one female subject had a difference between the ARI_base_ and ARI_apex_ of −39 ms, which was found in close proximity to the data points from simulations in the models with inverted ABRGs and arrhythmias. The close proximity of the ECGI and simulation data points in Fig. 6*B* illustrates that the apicobasal dispersion of ARI that was found in some normal human subjects was very similar to the apicobasal dispersion of APD in the computational models with arrhythmias, thereby suggesting the likelihood of increased arrhythmia vulnerability in these subjects.

## Discussion

Using previously acquired ECGI data, this study reveals a high variability of the ABRG in the ventricles of human subjects with no apparent ECG abnormality and shows that the ABRG tends to diminish with ageing. Computational simulations in human models of ventricular epicardium demonstrate that a diminished or inverted ABRG resulted in decreased arrhythmia sustainability, suggesting that the age‐related changes in ABRG can possibly lead to a decreased vulnerability to ventricular arrhythmia.

### The orientation of ABRG in normal human subjects and its dependence on age

The most common ABRGs observed in this study, in both males and females, had RT_ECGI,apex_ < RT_ECGI,base_ and ARI_apex_ < ARI_base_. This observation is in line with a previous ECGI study that characterized repolarization gradients in the human ventricles and reported ARI dispersion from the apex and base between 30 and 52 ms (Ramanathan et al., 2006). In our study, we observed several instances of ARI dispersion within this range. Though some subjects had asmaller ARI dispersion, and a few cases had an inverted ABRG, i.e. RT_ECGI,apex_ > RT_ECGI,base_ and ARI_apex_ > ARI_base_. The correlation analysis revealed that RT_ECGI,apex_, but not RT_ECGI,base_, is associated with the ABRG_ECGI_, suggesting that the presence of an inverted ABRG is caused by regional changes in RT_ECGI_, particularly by RT_ECGI,apex_ prolongation.

The negative association between ABRG_ECGI_ and age revealed in this study indicates that the apicobasal dispersion of ventricular repolarization is, at least to some extent, related to ageing. In subjects <50 years of age, only natural ABRGs were observed, i.e. RT_ECGI,apex_ < RT_ECGI,base_ and ARI_apex_ < ARI_base_. However, a large variety of ABRGs was found in older individuals, including ABRGs with RT_ECGI,apex_ > RT_ECGI,base_ and ARI_apex_ > ARI_base_. It is therefore likely that these differences are associated with age‐related changes in ventricular repolarization that lead to QT interval prolongation (Rabkin et al., 2016) and, possibly, with age‐related structural remodelling of the human ventricles (Grassow et al., 2025).

### Possible mechanistic explanation for the age‐related differences in ABRG

It is feasible that the age‐related differences in ABRG observed in this study stem from the regional effects of β‐adrenergic receptor stimulation (β‐ARS), which has been shown to promote heterogeneity in ventricular repolarization (Caldwell et al., 2023). Previous studies have demonstrated that β‐ARS substantially increases *I*_Ks_ activation (Sanguinetti et al., 1991; Volders et al., 2003) and that increased *I*_Ks_ in the presence of β‐ARS leads to more pronounced transmural dispersion of repolarization (Shimizu & Antzelevitch, 1998; Viswanathan et al., 1999). Given that *I*_Ks_ density is approximately double in apical cardiomyocytes compared to basal cardiomyocytes (Szentadrassy et al., 2005), it is plausible that greater β‐ARS at faster heart rates leads to larger apicobasal difference in *I*_Ks_ function and increases the apicobasal dispersion of ventricular repolarization. Since ageing is associated with a decline in resting heart rate and decreased β‐ARS responsiveness (White et al., 1994), the apicobasal difference in *I*_Ks_ possibly diminishes with ageing, leading to the gradual disappearance of ABRG. This mechanistic explanation is in line with our findings that show shorter repolarization times in the ventricular apex (RT_ECGI,apex_) of younger subjects (Fig. 3*B*) and at faster heart rates (Fig. 3*F*), and the decline in ABRG with increasing age (Fig. 3*A*) and at slower heart rates (Fig. 3*E*).

### ABRG and ventricular arrhythmia vulnerability

The computer simulations performed in this study demonstrated that steep ABRGs observed in normal human subjects can increase the vulnerability to ventricular arrhythmias and might, therefore, represent a ventricular arrhythmogenic substrate. The combination of ECGI‐derived and simulation data shows that natural ABRGs are not only more prevalent in normal human subjects, but that they can sustain arrhythmia for longer periods of time than inverted ABRGs.

Our observations that steep ABRGs increase arrhythmia vulnerability agree with the widely accepted notion that the presence of increased repolarization heterogeneity, including a steep repolarization gradient, is a prerequisite for the initiation of re‐entry and the occurrence of arrhythmia (Chauhan et al., 2006; Coronel et al., 2009). Local repolarization heterogeneities have been shown to increase arrhythmia vulnerability and lead to the spontaneous occurrence of arrhythmias (Renard et al., 2024). These repolarization heterogeneities have also been shown to play a crucial role in drug‐induced torsades de pointes arrhythmias (Rivaud et al., 2021). Our work proposes that the repolarization heterogeneities that contribute to arrhythmogenic substrates in the human ventricles can span across large distances in the ventricular yocardium.

In comparison to the work by Rivaud et al. (2021) that also used a human model of ventricular epicardium to investigate arrhythmia vulnerability, in our study the computational models in which arrhythmias occurred showed longer RT differences between the region of long and short RT (>121 *vs*. >70 ms). This difference is caused by the anatomical distances between the regions of short and long RT. In the study by Rivaud et al. (2021), the repolarization heterogeneity was induced by local infusion of the potassium current inhibitor sotalol, hence the RT gradient spanned over a distance of a few centimetres. However, in our study, the ABRG spans over the whole apicobasal distance, which was ∼9 cm in both models. If expressed in milliseconds per centimetres (>13 ms/cm in this study), the gradients would show comparable steepness.

### ABRG as a possible contributor to the arrhythmogenic substrate in idiopathic VF and sudden cardiac death

The presence of steep natural ABRGs in subjects <50 years of age and the occurrence of arrhythmias in the computational models that incorporated such ABRGs offer a hypothesis that steep natural ABRGs could contribute to the arrhythmogenic substrate in individuals who have experienced idiopathic VF or have suffered sudden cardiac death. Abnormal dispersion of ventricular repolarization has been found in idiopathic VF survivors, and it has been suggested that VF emerges from the interaction of a premature beat and the region with abnormally dispersed repolarization (Cluitmans et al., 2021). Studies on sudden arrhythmic death syndrome (SADS) reported a mean age of 31.4 years (Mellor et al., 2014), 32 years (Behr et al., 2007) and a median age of 24 years (Lahrouchi et al., 2017) in the SADS victims. This indicates that the majority of cases occurred within the age range where we observed steep natural ABRGs. Moreover, a gradual decrease in the number of SADS cases can be observed after 40 years of age (Mellor et al., 2014), which corresponds to our finding of ABRGs decline with increasing age, further supporting the potential association between ABRG and SADS. To unravel the possible role of ABRG in SADS and idiopathic VF, studying the interaction of ABRG and the Purkinje network would be of particular interest, because Purkinje ectopy has been documented as a frequent arrhythmia trigger in idiopathic VF survivors (Haïssaguerre et al., 2020).

In contrast to natural ABRGs, the presence of diminished or inverted ABRGs in the models was associated with fewer arrhythmias and shorter arrhythmia durations. These findings suggest that the diminishing and inversion of an ABRG with age could possibly lead to lower arrhythmogenicity. The dispersion of ventricular repolarization associated with inverted ABRG could decrease arrhythmia inducibility by attenuating the effect of arrhythmic triggers that originate from the apical portion of the ventricles (Haïssaguerre et al., 2020). From this perspective, less steep ABRG in subjects >50 years of age could be considered as an anti‐arrhythmic factor. However, should the triggers or the sites susceptible to APD alternans (Orini et al., 2019) be located at the basal portion of the ventricles, inverted ABRG could represent an important contributor to the arrhythmogenic substrate. Additional studies shouldbe performed to unravel the prevalence and electrophysiological relevance of inverted ABRGs in human ventricles.

### Sex‐specific differences in ABRGs and their role in arrhythmia vulnerability

The ECGI data presented in this study suggest generally higher variability in the dispersion of ventricular repolarization in females than in males. Indeed, inverted ABRGs were observed mainly in female subjects >50 years of age, whereas male subjects predominantly showed natural ABRGs. This sex‐specific difference in ABRG variability might be influenced, to some extent, by the disproportion between the number of female and male subjects in our cohort, but it could be also explained by the effects of sex hormones on ventricular electrophysiology.

It is feasible that the higher occurrence of inverted ABRGs in females >50 years of age is related to postmenopausal hormonal changes. Several ion currents involved in human ventricular repolarization have been shown to be affected by the presence of female sex hormones (Pham & Rosen, 2002). It has been shown that *I*_Ks_ in apical cardiomyocytes has approximately twice the current density of basal cardiomyocytes (Szentadrassy et al., 2005) and could therefore contribute to the age‐related differences in the ABRG observed in our study. Another ion current that could affect the apicobasal dispersion of ventricular repolarization is the L‐type Ca^2+^ current (*I*_CaL_). Experiments in adult and prepubertal rabbits have shown that *I*_CaL_ density in the basal cardiomyocytes of adult females is increased compared to adult males, and that sex steroids can modulate the apicobasal *I*_CaL_ distribution (Sims et al., 2008). The same study also demonstrated that distinct apicobasal distributions of *I*_CaL_ in males and females contribute to different arrhythmia phenotypes, with more frequent torsade de pointes arrhythmias in females.

The same number of arrhythmia occurrences was observed in the male and the female models, suggesting that the presence of a steep repolarization gradient in the ventricles can possibly outweigh the role of sex‐specific ventricular electrophysiology on arrhythmia vulnerability. In contrast, the arrhythmias induced by apical pacing lasted longer in the male model than in the female model, indicating higher arrhythmia sustainability in males in the presence of an equivalent dispersion of ventricular repolarization as in females. This effect was probably caused by longer APD in the female model that prolongs arrhythmia wavelength, acting as a safety factor, and by a larger surface area of the epicardial mesh in the male model that allowed for more simultaneously occurring re‐entrant circuits. To unravel fully the contribution to arrhythmia initiation and maintenance from the ABRG, sex‐specific electrophysiology, cardiac dimensions and, possibly other factors that were not included in our models, a more extensive study should be performed with patient‐specific models developed for a wider range of patients.

### Limitations

ECGI acquisitions were performed in a relatively small number of subjects, with an unequal representation of male and female subjects. As a consequence, the ECGI data might not fully capture the ABRG variability in a healthy adult human population, especially in males. A study in a bigger cohort of both healthy and unhealthy patients is warranted to fully characterize ABRG variability and to investigate whether inverted ABRGs occur with a similar frequency in male and female populations. To investigate further how the ABRG develops with increasing age and to unravel the possible effects of changes in sex hormone levels, a longitudinal study with multiple time points per subject would be necessary.

By using ECGI recordings acquired during a resting heart rate, our study did not allow for us to fully investigate the effect of heart rate on the ABRG. For example, the lack of information about the role of β‐ARS in our patient cohort prevented us from using a more complex computational approach that would have allowed us to incorporate the impact of β‐ARS on the ABRG (Heijman et al., 2011). Thus, future studies should focus on the rate dependence of the ABRG, as suggested by our data (Fig. 4*E*), with a particular emphasis on the potential role of β‐ARS when patient data are available.

ECGI data were used for this study because the method  non‐invasively maps ventricular electrophysiology. Although invasive mapping with catheters or electrode arrays provides higher accuracy (Cluitmans et al., 2017), it would have been unethical, expensive and unnecessarily risky to perform in non‐diseased human subjects.

Given that no ventricular arrhythmias were documented in the investigated subjects and that arrhythmias were observed only in the computational models, the study provides only indirect evidence for steep ABRGs promoting vulnerability to ventricular arrhythmia. ECGI data from a large cohort of patients with previously documented ventricular arrhythmias would help to assess directly the contribution of the ABRG to the ventricular arrhythmogenic substrate.

To assess the effects of a wide range of ABRGs and to investigate the effects of sex‐specific ventricular electrophysiology, human epicardial models were chosen for the computational part of the study. Previous studies have shown a good predictive value of epicardial models for studying ventricular arrhythmias in relation to steep repolarization gradients (Moreno et al., 2011; Rivaud et al., 2021).The presented observations in human epicardial models are also in line with our earlier work that demonstrated prolonged arrhythmia duration in the presence of a steep ABRG in a biventricular female model (Sobota et al., 2022). It is, therefore, plausible that replicating the computational part of the study with complete biventricular models would provide only a small increase in added value, while requiring considerably larger computational resources.

We defined the ABRG as the difference in recovery times (RT_ECGI_; ECGI data, ABRG_ECGI_), or its analog, repolarization times (RT_sim_; simulation data, ABRG_sim_). This definition aligns well with the approaches used in previous studies that identified steep repolarization gradients as a key part of a ventricular arrhythmogenic substrate (Cluitmans et al., 2021; Coronel et al., 2009; Rivaud et al., 2021). Similar results could be obtained for the ABRG_ECGI_ if the gradient was defined as the apicobasal difference in ARIs (i.e. ARI_base_ − ARI_apex_). However, this is not the case for the simulation data, because the models were paced either from the apex or from the RVOT, which does not emulate sinus rhythm and leads to considerable differences between RT_sim,RVOT_ and APD_RVOT_ during apical pacing and between RT_sim,apex_ and APD_apex_ during RVOT pacing. From this perspective, Fig. 6*B*, which visualizes ECGI and simulation data by plotting ARIs with APDs, compares the gradients more fairly than Fig. 6*A*, which shows ABRG_ECGI_ and ABRG_sim_ obtained from RT_ECGI_ and RT_sim_, respectively.

### Conclusion

A high variability in the ABRG is present in individuals with no apparent abnormality on the clinical 12‐lead ECG. The ABRG diminishes with ageing in both male and female human ventricles. Steep ABRGs observed in human subjects <50 years of age can increase ventricular arrhythmia vulnerability and might, therefore, contribute to the arrhythmogenic substrate in human ventricles. In contrast, the less steep ABRGs found in human subjects >50 years of age were associated with fewer arrhythmias, and their presence could possibly be considered as anti‐arrhythmic.

## Additional information

## Competing interests

M.J.M.C. is employed part time by Philips Research. All remaining authors declare that they have no competing interests.

## Author contributions

V.S. and J.D.B. designed the computational part of the study. J.S., K.H.K.P., F.S.N., P.G.A.V. and M.J.M.C. acquired the ECGI data. V.S. performed the simulations and analysed the data. V.S., J.S., R.S., H.N., E.G., F.S.N., P.G.A.V., M.J.M.C. and J.D.B. interpreted the data. V.S. and J.D.B. drafted the manuscript. All authors revised the manuscript critically for important intellectual content. All authors have read and approved the final version of the manuscript and agree to be accountable for all aspects of the work in ensuring that questions related to the accuracy or integrity of any part of the work are appropriately investigated and resolved. All persons designated as authors qualify for authorship, and all those who qualify for authorship are listed as authors.

## Funding

This research was funded by the French National Research Agency grant ANR‐10‐IAHU‐04 (V.S. and J.D.B.), by Fondation Lefoulon‐Delalande (V.S.), the France‐Berkeley Fund (E.G. and J.D.B.), the Netherlands CardioVascular Research Initiative (CVON2017‐13 VIGILANCE), the British Heart Foundation grants RG/F/22/110078 and RE/24/130023 (F.S.N.), the National Institute for Health Research Imperial Biomedical Research Centre (F.S.N.), the Institute of International Education Quad Fellowship (R.S.), the American Heart Association Career Development Award 24CDA1258695 (H.N.) and the NHLBI Grants R01HL131517 (E.G.), P01HL141084 (E.G.) and R01HL170521 (E.G. and H.N.). The project was provided with high‐performance computing and storage resources by Grand Équipement National de Calcul Intensif (GENCI) at Très Grand Centre de Calcul (TGCC) thanks to the grant 2024‐A0160310517 on the supercomputer Joliot Curie's ROME partition (V.S. and J.D.B).

## Supporting information


Peer Review History

## Data Availability

All relevant data are provided within the manuscript. The computational models are available upon request to the corresponding author.
