# Supplementary material for: The apicobasal dispersion of ventricular repolarization in humans is associated with age and affects arrhythmia vulnerability
Source: J Physiol. 2025 May 28;604(13):5260–75. doi: 10.1113/JP288356 (PMC12288923; doi:10.1113/JP288356)
Supplement: Supplementary file 1 — Peer Review History [file TJP-604-5260-s001.pdf]

The Journal of Physiology

The apicobasal dispersion of ventricular repolarization in humans is associated with age and affects arrhythmia vulnerability

Vladimír Sobota, Job Stoks, Kiran Haresh Kumar Patel, Roshni Shetty, Haibo Ni, Eleonora Grandi, Fu Siong Ng, Paul GA Volders, Matthijs J.M. Cluitmans, and Jason D Bayer
DOI: 10.1113/JP288356

Corresponding author(s): Vladimír Sobota (vladimir.sobota@ihu-liryc.fr)

The referees have opted to remain anonymous.

Review Timeline:

Submission Date:	18-Dec-2024
Editorial Decision:	14-Feb-2025
Revision Received:	14-Mar-2025
Editorial Decision:	27-Mar-2025
Revision Received:	31-Mar-2025
Accepted:	01-Apr-2025

Senior Editor: Bjorn Knollmann

Reviewing Editor: Bjorn Knollmann

Transaction Report:

(Note: With the exception of the correction of typographical or spelling errors that could be a source of ambiguity, letters and reports are not edited. Depending on transfer agreements, referee reports obtained elsewhere may or may not be included in this compilation. Referee reports are anonymous unless the Referee chooses to sign their reports.)

Dear Dr Sobota,

Re: JP-RP-2024-288356 "The apicobasal dispersion of ventricular repolarization in humans is associated with age and affects arrhythmia vulnerability" by Vladimír Sobota, Job Stoks, Kiran Haresh Kumar Patel, Roshni Shetty, Haibo Ni, Eleonora Grandi, Fu Siong Ng, Paul GA Volders, Matthijs J.M. Cluitmans, and Jason D Bayer

Thank you for submitting your manuscript to The Journal of Physiology. It has been assessed by a Reviewing Editor and by 1 expert referee and we are pleased to tell you that it is acceptable for publication following satisfactory revision.

The referee reports are copied at the end of this email.

Please address all the points raised and incorporate all requested revisions or explain in your Response to Referees why a change has not been made. We hope you will find the comments helpful and that you will be able to return your revised manuscript within 4 weeks. If you require longer than this, please contact journal staff: jp@physoc.org.

Your revised manuscript should be submitted online using the link in your Author Tasks Link Not Available. This link is accessible via your account as Corresponding Author; it is not available to your co-authors. If this presents a problem, please contact journal staff (jp@physoc.org). Image files from the previous version are retained on the system. Please ensure you replace or remove any files that are being revised.

If you do not wish to submit a revised version of your manuscript, you must inform our journal staff (jp@physoc.org) or reply to this email to request withdrawal. Please note that a manuscript must be formally withdrawn from the peer review process at one journal before it may be submitted to another journal.

TRANSPARENT PEER REVIEW POLICY: To improve the transparency of its peer review process The Journal of Physiology publishes online, as supporting information, the peer review history of all articles accepted for publication. Readers will have access to decision letters, including Editors' comments and referee reports, for each version of the manuscript, as well as any author responses to peer review comments. Referees can decide whether or not they wish to be named on the peer review history document.

ABSTRACT FIGURES: Authors are expected to use The Journal's premium BioRender account to create/redraw their Abstract Figures. Information on how to access this account is here: https://physoc.onlinelibrary.wiley.com/journal/14697793/biorender-access.

This will enable Authors to create and download high-resolution figures. If authors have used the free BioRender service, they can use the instructions provided in the link above to download a high-resolution version suitable for publication.
The link provided should only be used for the purposes of this submission. Authors will be charged for figures created on this account if they are not related to this manuscript submission.

LANGUAGE EDITING AND SUPPORT FOR PUBLICATION: If you would like help with English language editing, or other article preparation support, Wiley Editing Services offers expert help, including English Language Editing, as well as translation, manuscript formatting, and figure formatting at www.wileyauthors.com/eeo/preparation. You can also find resources for Preparing Your Article for general guidance about writing and preparing your manuscript at www.wileyauthors.com/eeo/prepresources.

RIGOUR AND REPRODUCIBILITY: We recommend authors consult The Journal's Rigour and Reproducibility requirements to ensure that their manuscript is suitable for publication: https://physoc.onlinelibrary.wiley.com/pb-assets/hub-assets/physoc/documents/TJP-Rigour-and-Reproducibility-Requirements.pdf

REVISION CHECKLIST:
Check that your Methods section conforms to journal policy: https://jp.msubmit.net/cgi-bin/main.plex?form_type=display_requirements#methods

Check that data presented conforms to the statistics policy: https://jp.msubmit.net/cgi-bin/main.plex?form_type=display_requirements#statistics

Upload a full Response to Referees file. To create your 'Response to Referees' copy all the reports, including any comments from the Senior and Reviewing Editors, into a Microsoft Word, or similar, file and respond to each point, using font or background colour to distinguish comments and responses and upload as the required file type.

Please upload two versions of your manuscript text: one with all relevant changes highlighted and one clean version with no changes tracked. The manuscript file should include all tables and figure legends, but each figure/graph should be uploaded as separate, high-resolution files. The journal is now integrated with Wiley's Image Checking service. For further details, see: https://www.wiley.com/en-us/network/publishing/research-publishing/trending-stories/upholding-image-integrity-wileys-image-screening-service

You may also upload:
- 'Potential Cover Art' for consideration as the issue's cover image
- Appropriate Supporting Information (Video, audio or data set: see https://jp.msubmit.net/cgi-bin/main.plex?form_type=display_requirements#supp).

We look forward to receiving your revised submission.

If you have any queries, please reply to this email and we will be pleased to advise.

Yours sincerely,

Bjorn Knollmann
Senior Editor
The Journal of Physiology

REQUIRED ITEMS

- Author photo and profile. First or joint first authors are asked to provide a short biography (no more than 100 words for one author or 150 words in total for joint first authors) and a portrait photograph. These should be uploaded and clearly labelled together in a Word document with the revised version of the manuscript. See Information for Authors for further details.

- You must start the Methods section with a paragraph headed Ethical Approval. If experiments were conducted on humans, confirmation that informed consent was obtained, preferably in writing, that the studies conformed to the standards set by the latest revision of the Declaration of Helsinki and that the procedures were approved by a properly constituted ethics committee, which should be named, must be included in the article file. If the research study was registered (clause 35 of the Declaration of Helsinki), the registration database should be indicated, otherwise the lack of registration should be noted as an exception (e.g. The study conformed to the standards set by the Declaration of Helsinki, except for registration in a database). For further information see: https://physoc.onlinelibrary.wiley.com/hub/human-experiments.

- Your manuscript must include a complete Additional Information section, including competing interests; funding; author contributions and acknowledgements.

- Please upload separate high-quality figure files via the submission form.

- Please include an Abstract Figure file, as well as the Figure Legend text within the main article file. The Abstract Figure is a piece of artwork designed to give readers an immediate understanding of the research and should summarise the main conclusions. If possible, the image should be easily 'readable' from left to right or top to bottom. It should show the physiological relevance of the manuscript so readers can assess the importance and content of its findings. Abstract Figures should not merely recapitulate other figures in the manuscript. Please try to keep the diagram as simple as possible and without superfluous information that may distract from the main conclusion(s). Abstract Figures must be provided by authors no later than the revised manuscript stage and should be uploaded as a separate file during online submission labelled as File Type 'Abstract Figure'. Please also ensure that you include the figure legend in the main article file. All Abstract Figures should be created using BioRender. Authors should use The Journal's premium BioRender account to export high-resolution images. Details on how to use and access the premium account are included as part of this email.

EDITOR COMMENTS

Reviewing Editor:

Thank you for the manuscript. I agree with the referee that this is a great manuscript. However, there are certain statements that could be refined to ensure they are not overly assertive.

Please also see 'Required Items' above.

Senior Editor:

I concur with the reviewing editor. This is potentially impactful work, but will require addressing the concerns raised by the reviewer before it can be accepted.

REFEREE COMMENTS

Referee #1:

In this manuscript, Sobota and colleagues combine population data and human computational modelling and simulation to study the effect of age and sex on the ventricular apicobasal repolarization gradient. The manuscript is well written, clear and demonstrates numerous scientific insights. Therefore, the following points are only minor concerns I would like to discuss with the authors:

1. I wonder whether it is fair to compute the apicobasal repolarization gradient (ABRG) from the ECGi and the simulations in the same way and only as a function of the recovery time (RT). During sinus rhythm, due to the Purkinje network, the myocardium is almost simultaneously depolarized. If we consider a theoretical case in which all cells are depolarized at the same time, then ABRG=ΔRT=ΔAPD. This is in agreement with the ECGi data of this study, in which the ABRG (computed as ΔRT) is similar to ΔARI (surrogate of ΔAPD). In the simulations, however, the authors have not used a Purkinje network or paced the epicardial surface simultaneously, so ΔRT= (ΔAPD + depolarization time from apex to base). Therefore, if the authors define ABRG=ΔRT, the conduction velocity and the dimensions of the mesh employed might have a huge impact on ABRG, even more than ΔAPD. This explains why, in Figure 6A, ABRG_ECGi and ABRG_sim do not overlap (but APD_sim and ARI_ECGi do) or why the bigger male mesh sustains longer arrhythmia episodes despite ΔAPD being longer in females. In my view, the conclusion in Lines 270-272 (ABRG_ECGI observed in healthy subjects during sinus rhythm were not steep enough to lead to arrhythmias) is influenced by an unfair comparison between ABRG_ECGi (computed in sinus rhythm) and ABRG_sim (computed using a pacing protocol from the apex/base). If ABRG would have been defined as ABRG = ΔARI (or ABRG = ΔAPD) in both ECGi and simulations, then the ABRG_ECGI observed in healthy subjects during sinus rhythm would be, indeed, steep enough to cause arrhythmias, as shown in Figure 6B. What do the authors think?

2. This small discrepancy between ECGi and simulations brings me to another point. The approach of combining population data and human computational modelling and simulation is scientifically sound. However, while reading the manuscript I had the impression that both parts (i.e., human data and simulations) are presented as independent sub-studies and only compared at the end. I was surprised to see that none of the conclusions obtained from human ECGi has been used to inform the modelling approach. For example:

2.1. The study shows the same arrhythmia inducibility between genders. This is different from Sobota et al. 2022, were the longer APD and steeper repolarization gradient in the female model resulted in a higher proarrhythmia. Similar to Sobota et al. 2022, the population data in this study seem to indicate that, at least under 50 years old, ARI are longer in women than in men (40 [30-45] ms in females vs. 32 [27-38] ms in male). Therefore, while I acknowledge that 22 subjects might not be enough to constrain the models in a specific way, I wonder whether the authors thought of using steeper gradients in the female model.

2.2. In Line 388-389, the authors suggest "a limited role of sex-specific ventricular electrophysiology on arrhythmia inducibility in the presence of a steep ABRG". This is in contrary to experimental data from rabbits, as the authors discuss. What about humans? Is there human data to support a limited role of sex-specific ventricular electrophysiology on arrhythmogenesis that the authors could include in the discussion? What can the authors really conclude when only one mesh per sex has been used, the same tissue connectivity, and the same scaling factors for potassium currents are imposed in both sex models, despite the human ECGi data implying a steeper gradient in women (or at least a greater ΔARI). With the methodological setup employed by the authors and their definition of ABRG it is very difficult to tell what is favouring proarrhythmia: ΔAPD (greater in the female model) or a bigger mesh (greater in the male model). I am not going to ask the authors to run more simulations with additional meshes, tissue conductivities and considering sex-specific differences in ABRG, but I believe they should indicate that a more extensive simulation study might be needed to make a solid statement.

2.3. In my view, there is a very convincing mechanistic explanation between resting rate, ABRG and ageing that the authors have not emphasized enough, and definitely not included in the models. IKs is a key sarcolemmal target of the β-adrenergic receptor stimulation (β-ARS). Activation of protein kinase A through β-ARS phosphorylates IKs, enhancing its activity. In terms of beating rate, increased IKs activation will derive from faster rates, which are associated with a greater β-ARS. Additionally, since the density of IKs is higher in the apex compared to the ventricular base, β-ARS results in a more pronounced apicobasal difference in IKs function. This difference arises primarily from the heightened activity in the apex under β-ARS, and explains the steeper ABRG observed at faster resting rates (Figure 3E) and the clear association with the recovery time in the apex (Figure 3F) but not the base (Table 1). Moreover, it could explain why a lower resting rate or ageing, and the loss-of-function of β-ARS, tend to equalize IKs activity in the apex and the base, eventually neutralizing ABRG (Figure 3A and 3E). I think that the authors present a lot of evidence that deserves a deeper discussion than the simple sentence (Line 313 - 314) dedicated in the discussion to the role of β-ARS and ageing on ABRG. Similarly, this functional phenomenon of beating rate and ABRG observed in the ECGi data is not translated to the models. In the simulations, the ABRG is derived from the S1 stimulation (i.e., slow pacing). Is the ABRG exacerbated at fast S2-S3 pacing according to the human ECGi data? Does it follow the same trend? In my view, the authors could use this human data to inform or validate the models, unifying both parts.

3. The idea that advanced age could be cardioprotective (regarding ABRG) is very interesting, since it opposes to the natural intuition of ageing and increased cardiovascular risk. I like that the authors linked steep natural ABRGs to idiopathic VF, but could they provide further evidence that idiopathic ventricular arrhythmias are more common in younger individuals to strengthen their finding that steep ABRG might be more responsible for arrhythmias in subjects <50 years of age, compared to older individuals in which other factors (e.g., fibrosis) could play a greater role.

4. I would appreciate if the authors indicated the conduction velocities of the tissue, or the sigma values used to achieve these velocities. The meshes appear to be slightly coarse, with some elements likely exceeding 500 microns of edge length. Thus, stating the velocity values might reassure the reader that those situations of multiple simultaneous re-entrant circuits, among others, are not the result of convergence errors.

5. In Line 153, the authors state that they have computed 112 simulations in total. Did the authors mean 112 simulations in each model (224 in total)? There are 56 apicobasal gradients, a male and a female model, and two stimulation locations.

END OF COMMENTS

Response to Reviewers

We thank the reviewer and editors for their constructive feedback. For clarity, we have colored the reviewer's comments in blue, added/edited text in the manuscript in red, original manuscript text in gray, and the text of the rebuttal in black.

Editor Comments

Reviewing Editor: Thank you for the manuscript. I agree with the referee that this is a great manuscript. However, there are certain statements that could be refined to ensure they are not overly assertive.
We thank the Reviewing Editor for their positive feedback. We have rephrased several sections of the manuscript. The detailed description of all edits is provided below.

Senior Editor: I concur with the reviewing editor. This is potentially impactful work, but will require addressing the concerns raised by the reviewer before it can be accepted.
We thank the Senior Editor for their positive comment. Based on the reviewer's feedback, we made several modifications in the manuscript, as described below. These revisions have hopefully addressed all of the concerns raised by the reviewer.

Referee Comments

In this manuscript, Sobota and colleagues combine population data and human computational modelling and simulation to study the effect of age and sex on the ventricular apicobasal repolarization gradient. The manuscript is well written, clear and demonstrates numerous scientific insights. Therefore, the following points are only minor concerns I would like to discuss with the authors:

1. I wonder whether it is fair to compute the apicobasal repolarization gradient (ABRG) from the ECGi and the simulations in the same way and only as a function of the recovery time (RT). During sinus rhythm, due to the Purkinje network, the myocardium is almost simultaneously depolarized. If we consider a theoretical case in which all cells are depolarized at the same time, then ABRG=ΔRT=ΔAPD. This is in agreement with the ECGi data of this study, in which the ABRG (computed as ΔRT) is similar to ΔARI (surrogate of ΔAPD). In the simulations, however, the authors have not used a Purkinje network or paced the epicardial surface simultaneously, so ΔRT= (ΔAPD + depolarization time from apex to base). Therefore, if the authors define ABRG=ΔRT, the conduction velocity and the dimensions of the mesh employed might have a huge impact on ABRG, even more than ΔAPD. This explains why, in Figure 6A, ABRG_ECGi and ABRG_sim do not overlap (but APD_sim and ARI_ECGi do) or why the bigger male mesh sustains longer arrhythmia episodes despite ΔAPD being longer in females. In my view, the conclusion in Lines 270-272 (ABRG_ECGI observed in healthy subjects during sinus rhythm were not steep enough to lead to arrhythmias) is influenced by an unfair comparison between ABRG_ECGi (computed in sinus rhythm) and ABRG_sim (computed using a pacing protocol from the apex/base). If ABRG would have been defined as ABRG = ΔARI (or ABRG = ΔAPD) in both ECGi and simulations, then the ABRG_ECGI observed in healthy subjects during sinus rhythm would be, indeed, steep enough to cause arrhythmias, as shown in Figure 6B. What do the authors think?
We thank the reviewer for their comment and we fully agree with them. Whether to calculate the apicobasal repolarization gradient (ABRG) as the difference in recovery times (RTs) or activation-recovery intervals (ARIs) was a topic of debate when designing the data analysis and preparing the manuscript. In the end, we chose to define ABRG as the difference in RTs due to several reasons:

- Defining ABRG as the difference in RTs **aligns better with the intuitive understanding of ABRG being a gradient of repolarization/recovery** times.

- **Previous studies** that identified steep repolarization gradients as a key component of ventricular arrhythmogenic substrates (Coronel et al., 2009; Cluitmans et al., 2021; Rivaud et al., 2021) **calculated the gradient as the difference in repolarization times**. Obtaining ABRG as the difference in RTs is therefore closer to the approach used in these studies, and allows to directly compare the gradients, as we do in the Discussion:

Compared to the work by Rivaud et al. that also utilized a human model of ventricular epicardium to investigate arrhythmia vulnerability (Rivaud et al., 2021), in our study the computational models in which arrhythmias occurred showed longer RT differences between the region of long and short RT (>121 ms vs. >70 ms). This difference is caused by the anatomical distances between the regions of short and long RT. In the study by Rivaud et al. the repolarization heterogeneity was induced by local infusion of potassium current inhibitor sotalol, so the RT gradient spanned over a distance of a few centimeters. However, in our study the ABRG spans over the whole apicobasal distance, which was approximately 9 cm in both models. If expressed in milliseconds per centimeter (>13 ms/cm in this study), the gradients would show comparable steepness.

- As the reviewer correctly mentions, **obtaining ABRGs from RTs and ARIs leads to similar results, especially for gradients calculated from ECGI data**. To illustrate this similarity, we provide Figures S1 and S2, and Table S1 below. These figures and the table use ARIs instead of RTs, and represent alternative versions of Figures 2 and 3, and Table 1 from the manuscript, respectively. Since presenting the ECGI results as RTs produced similar figures as using ARIs, while also taking into account the two points stated above, we chose to present the results as RTs.
- **Presenting the ABRGs from the simulation data as the differences in repolarization times also led to results that look similar as if the gradient was defined as the difference in action potential durations (APDs)**. To illustrate that, we created an alternative version of Figure 4, provided below as Figure S3. The figure uses APDs instead of repolarization times, and panel S3B conveys the same message as Figure 4B in the manuscript. Moreover, such an approach allows for a direct comparison with the local drug-induced repolarization time differences reported in the study by Rivaud et al. (Figure 2C,E,F)(Rivaud *et al.*, 2021).

The reviewer is correct about the comparison between $ABRG_{ECGI}$ and $ABRG_{sim}$ presented in Figure 6A. To allow the reader to see the difference between ABRGs based on RTs and repolarization times, and the gradients that are based on differences in ARIs and APDs, Figure 6 includes panel B. In the new version of the manuscript, we provide our reasoning for calculating ABRG in Limitations:

We defined ABRG as the difference in recovery times (RT_{ECGI}; ECGI data, $ABRG_{ECGI}$) or repolarization times (RT_{sim}; simulation data, $ABRG_{sim}$). Such definition aligns better with the intuitive understanding of ABRG being a repolarization gradient, and follows the approach used in previous studies that identified steep repolarization gradients as a key part of ventricular arrhythmogenic substrate (Coronel et al., 2009; Cluitmans et al., 2021; Rivaud et al., 2021). Similar results could be obtained for $ABRG_{ECGI}$ if the gradient was defined as the apicobasal difference in ARIs (ARI_{base} - ARI_{apex}). However, this is not the case for the simulation data, because the models were paced either from the apex or RVOT, leading to considerable differences between $RT_{sim,RVOT}$ and APD_{RVOT} during apical pacing, and $RT_{sim,apex}$ and APD_{apex} during RVOT pacing. From this perspective, Figure 6B that visualizes ECGI and simulation data by plotting ARIs with APDs compares the gradients more fairly than Figure 6A that shows $ABRG_{ECGI}$ and $ABRG_{sim}$ obtained from RT_{ECGI} and RT_{sim}, respectively.

In addition, we removed the text the reviewer was referring to (former lines 270-272 in Results), as this interpretation of the data could be seen as incorrect:

To identify ABRGs that occur in normal human subjects, as well as their association to an increase in arrhythmia vulnerability, the ECGI data were plotted together with the data from computer simulations with positive arrhythmia outcome. For repolarization times (Figure 6A), the ECGI and simulation data were located in non-overlapping clusters. ~~meaning that $ABRG_{ECGI}$ observed in healthy subjects during sinus rhythm were not steep enough to lead to arrhythmias that were observed in the presence of $ABRG_{sim}$ in the~~

~~*computational models*~~*. However, when apicobasal differences in ARIs and APDs were plotted together against* ARI_{apex} *and* APD_{apex}*, respectively (Figure 6B), data points from 6 female and 3 male subjects were found to be in close proximity of the data points from computer simulations with natural ABRGs in which arrhythmias occurred. …*

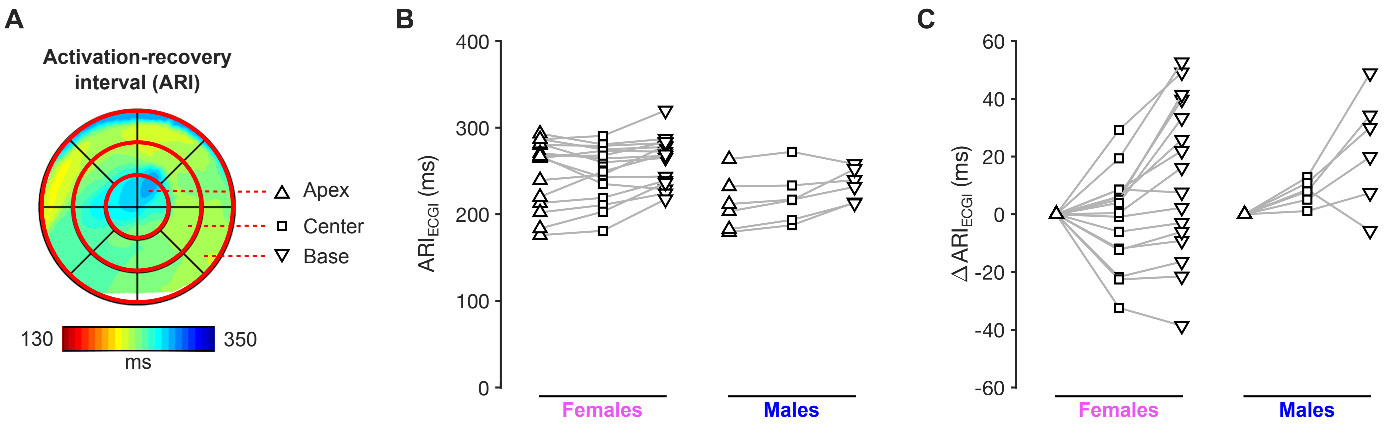

Figure S1. Apicobasal repolarization gradients determined from local activation-recovery intervals (ARI). (A) Local ARIs were obtained from the electrocardiographic imaging (ECGI) data, and average ARI was determined for the apical (ARI_{apex}), central and basal regions. **(B)** Average ARI for apical, central and basal regions, plotted independently for female and male subjects. **(C)** Differences in average ARIs with respect to ARI_{apex}. The bullseye plot example presented in panel A was adopted from the original ECGI study (Stoks *et al.*, 2024). **For comparison with the results obtained with recovery times, see Figure 2 in the manuscript.**

Table S1. Overview of parameter correlations

	Age	RR	$ABRG_{ECGI}$	ARI_{apex}
RR	r=0.4047	----	----	----
	p=0.0599			
$ABRG_{ECGI}$	r=-0.7140#	r=-0.4737#	----	----
	p=0.0002	p=0.0273		
ARI_{apex}	r=0.5788#	r=0.6928#	r=-0.7007#	----
	p=0.0048	p=0.0005	p=0.0004	
ARI_{base}	r=0.3225	r=0.7120	r=-0.2061#	r=0.7922
	p=0.1433	p=0.0003	p=0.3558	p<0.0001

$ABRG_{ECGI}$ – apicobasal repolarization gradient derived from electrocardiographic imaging (ECGI) data; ARI_{apex} – average activation-recovery time at the apex; ARI_{base} – average activation-recovery time at the base
Graph shown in Figure S2.
For comparison with the results obtained with recovery times, see Table 1 in the manuscript.

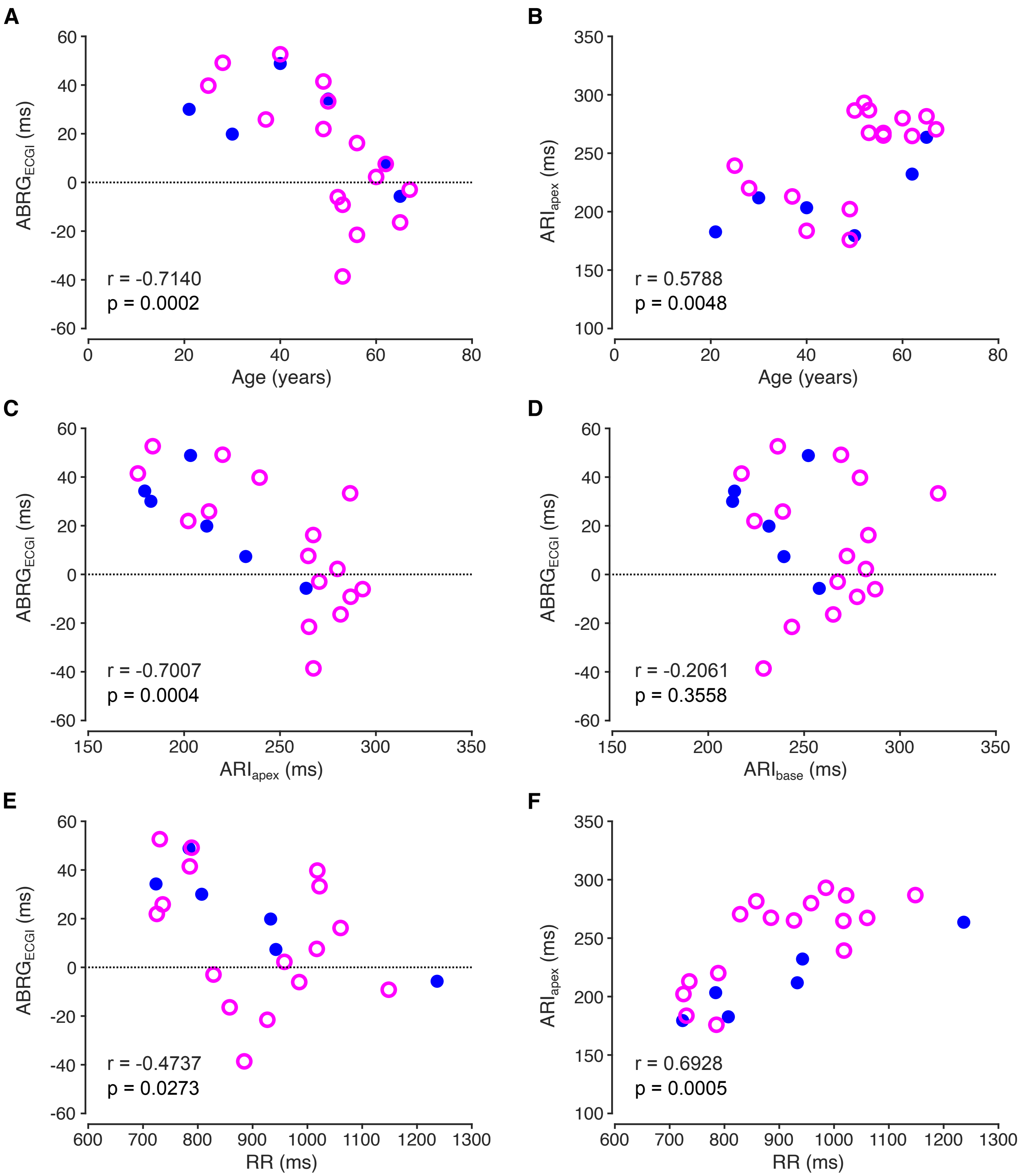

Figure S2. Apicobasal repolarization gradient determined from electrocardiographic imaging data ($ABRG_{ECGI}$) diminishes with age due to prolongation of activation-recovery intervals at the ventricular apex (ARI_{apex}). (A) A negative association was found between $ABRG_{ECGI}$ and age. **(B)** A positive association was found between average ARI_{apex} and age. **(C)** $ABRG_{ECGI}$ negatively correlated with ARI_{apex} but no association was found between $ABRG_{ECGI}$ and average activation-recovery interval at the base (ARI_{base})**(D)**. **(E)** $ABRG_{ECGI}$ was negatively associated with RR interval, indicating that steeper $ABRG_{ECGI}$ generally occurred at faster heart rates. **(F)** ARI_{apex} positively correlated with RR interval and the same behaviour was found for ARI_{base} (graph not shown). **For comparison with the results obtained with recovery times, see Figure 4 in the manuscript.**

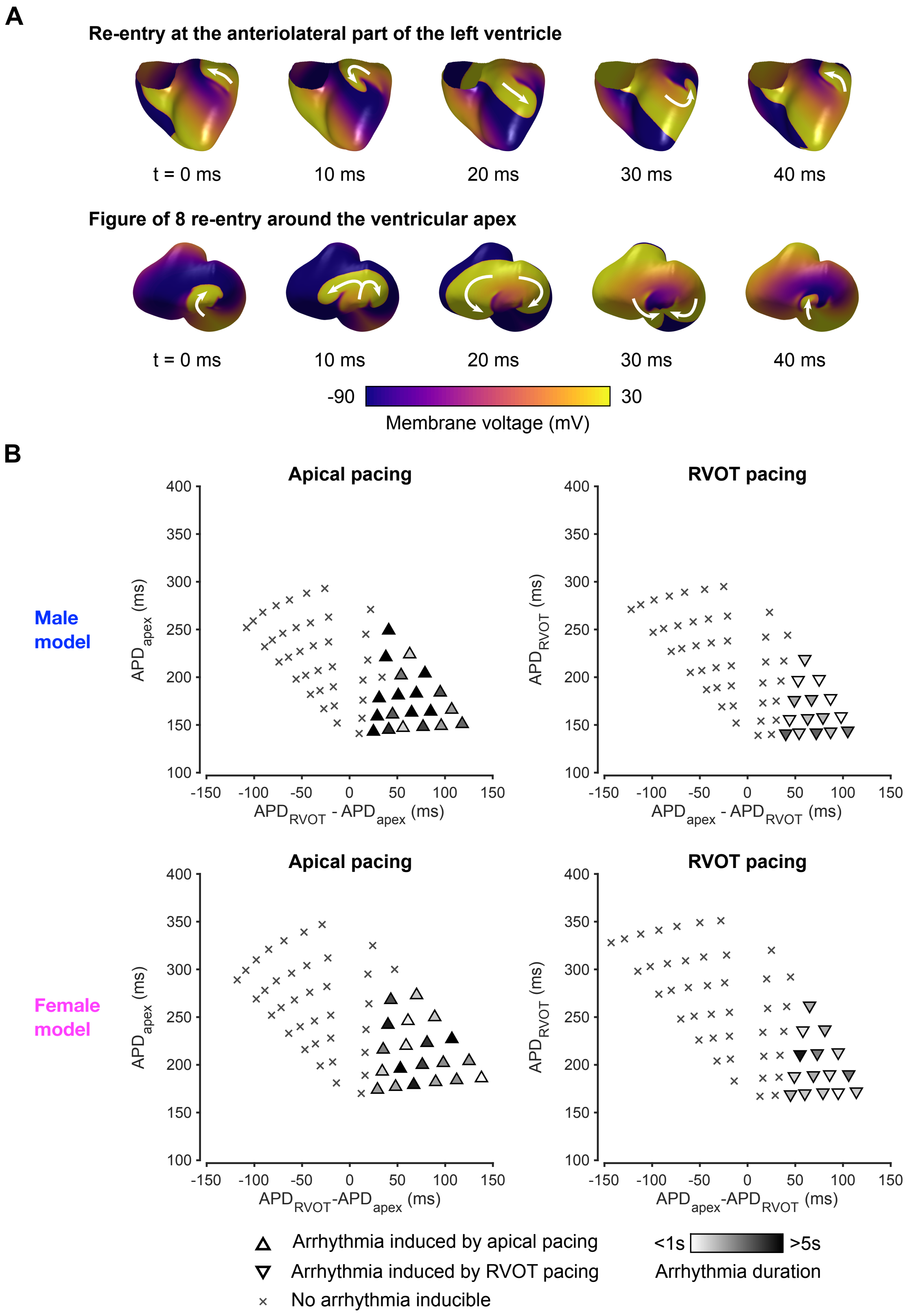

Figure S3. Arrhythmia inducibility in the computational models. (A) Examples of reentrant arrhythmias observed in the models. **(B)** Arrhythmia inducibility during apical and RVOT pacing in the male and the female model. **For comparison with the results obtained with repolarization times, see Figure 4 in the manuscript.**

2. This small discrepancy between ECGi and simulations brings me to another point. The approach of combining population data and human computational modelling and simulation is scientifically sound. However, while reading the manuscript I had the impression that both parts (i.e., human data and simulations) are presented as independent sub-studies and only compared at the end. I was surprised to see that none of the conclusions obtained from human ECGi has been used to inform the modelling approach. For example:

2.1. The study shows the same arrhythmia inducibility between genders. This is different from Sobota et al. 2022, were the longer APD and steeper repolarization gradient in the female model resulted in a higher proarrhythmia. Similar to Sobota et al. 2022, the population data in this study seem to indicate that, at least under 50 years old, ARI are longer in women than in men (40 [30-45] ms in females vs. 32 [27-38] ms in male). Therefore, while I acknowledge that 22 subjects might not be enough to constrain the models in a specific way, I wonder whether the authors thought of using steeper gradients in the female model.

The reviewer is correct. In our previous study (Sobota *et al.*, 2022), we showed higher arrhythmia vulnerability in a female model of human ventricles. However, there are methodological aspects we would like to point out when comparing the results of the current study with our previous work. First, only 2 different levels of natural ABRG were implemented in the previous study, while the current study uses 56 different ABRGs, both natural and inverted ones, which as a result tests a much wider range of ABRGs in each model. From this perspective, our previous study might represent rather an illustrative than a representative case, and in the context of our current work we refrain from drawing any strong conclusions from it.

Secondly, we chose not to model ABRG in a sex-specific manner exactly for the reasons the reviewer describes – the sample size was too small and the ABRG variability was too high to make a strong claim about ABRG being steeper in one of the sexes (possibly in females, as the data suggest). We acknowledge this aspect in Limitations:

> *ECGI acquisitions were performed in a relatively small number of subjects with an unequal representation of male and female subjects. As a consequence, the ECGI data may not fully capture the ABRG variability in healthy adult human population, especially in males. A study in a bigger cohort of both healthy and unhealthy patients is warranted to fully characterize the ABRG variability and to investigate whether inverted ABRGs occur with similar frequency in male and female populations.*

Given these limitations, we focused on studying the combined effect of different ABRGs with sex-specific electrophysiology and mesh geometry.

2.2. In Line 388-389, the authors suggest "a limited role of sex-specific ventricular electrophysiology on arrhythmia inducibility in the presence of a steep ABRG". This is in contrary to experimental data from rabbits, as the authors discuss. What about humans? Is there human data to support a limited role of sex-specific ventricular electrophysiology on arrhythmogenesis that the authors could include in the discussion? What can the authors really conclude when only one mesh per sex has been used, the same tissue connectivity, and the same scaling factors for potassium currents are imposed in both sex models, despite the human ECGi data implying a steeper gradient in women (or at least a greater ΔARI). With the methodological setup employed by the authors and their definition of ABRG it is very difficult to tell what is favouring proarrhythmia: ΔAPD (greater in the female model) or a bigger mesh (greater in the male model). I am not going to ask the authors to run more simulations with additional meshes, tissue conductivities and considering sex-specific differences in ABRG, but I believe they should indicate that a more extensive simulation study might be needed to make a solid statement.

We thank the reviewer for their insightful remark. To the best of our knowledge, there is no study that would provide strong evidence for a limited role of sex-specific ventricular electrophysiology on arrhythmogenesis. We agree with the reviewer that our methodological approach does not allow to quantify the contribution of the repolarization gradient, sex-specific electrophysiology, or cardiac dimensions on arrhythmia vulnerability.

Therefore, in line with the reviewer's comments, we have toned down the statement about sex-specific ventricular electrophysiology, and we mention that a more extensive study should be performed:

> *The same number of arrhythmia occurrences was observed in the male and the female models, suggesting that the presence of a steep repolarization gradient in the ventricles can possibly outweigh the role of sex-specific ventricular electrophysiology on arrhythmia vulnerability. On the other hand, the arrhythmias induced by apical pacing lasted longer in the male than the female models, indicating higher arrhythmia sustainability in males in the presence of equivalent dispersion of ventricular repolarization as in females. This effect was likely caused by longer APD in the female models that prolongs arrhythmia wavelength, acting as a safety factor, as well as by a larger epicardial mesh in the male model that allowed for more simultaneously occurring reentrant circuits. To fully unravel the contribution of ABRG, sex-specific electrophysiology, cardiac dimensions, and possibly other factors that were not included in our models, a more extensive study should be performed with patient-specific models developed for a wider range of patients.*

2.3. In my view, there is a very convincing mechanistic explanation between resting rate, ABRG and ageing that the authors have not emphasized enough, and definitely not included in the models. I_{Ks} is a key sarcolemmal target of the β-adrenergic receptor stimulation (β-ARS). Activation of protein kinase A through β-ARS phosphorylates I_{Ks}, enhancing its activity. In terms of beating rate, increased I_{Ks} activation will derive from faster rates, which are associated with a greater β-ARS. Additionally, since the density of I_{Ks} is higher in the apex compared to the ventricular base, β-ARS results in a more pronounced apicobasal difference in I_{Ks} function. This difference arises primarily from the heightened activity in the apex under β-ARS, and explains the steeper ABRG observed at faster resting rates (Figure 3E) and the clear association with the recovery time in the apex (Figure 3F) but not the base (Table 1). Moreover, it could explain why a lower resting rate or ageing, and the loss-of-function of β-ARS, tend to equalize I_{Ks} activity in the apex and the base, eventually neutralizing ABRG (Figure 3A and 3E). I think that the authors present a lot of evidence that deserves a deeper discussion than the simple sentence (Line 313 - 314) dedicated in the discussion to the role of β-ARS and ageing on ABRG. Similarly, this functional phenomenon of beating rate and ABRG observed in the ECGi data is not translated to the models. In the simulations, the ABRG is derived from the S1 stimulation (i.e., slow pacing). Is the ABRG exacerbated at fast S2-S3 pacing according to the human ECGi data? Does it follow the same trend? In my view, the authors could use this human data to inform or validate the models, unifying both parts.

We thank the reviewer for their excellent suggestion and offering a possible mechanistic explanation of the age-related differences in ABRG. We have rephrased the paragraph that previously contained lines 313-314 and added a section in which we provide the possible mechanistic explanation for the age-related differences in ABRG:

> *The negative association between $ABRG_{ECGI}$ and age revealed in this study indicates that the apicobasal dispersion of ventricular repolarization is, at least to some extent, related to aging. In subjects <50 years of age, only natural ABRGs were observed, i.e. $RT_{ECGI,apex} < RT_{ECGI,base}$ and $ARI_{apex} < ARI_{base}$. However, a large variety of ABRGs was found in older individuals, including the ABRGs with $RT_{ECGI,apex} > RT_{ECGI,base}$ and $ARI_{apex} > ARI_{base}$. It is therefore likely that these differences are associated with the age-related changes in ventricular repolarization that lead to QT interval prolongation (Rabkin et al., 2016), and possibly with the age-related structural remodeling of human ventricles (Grassow et al., 2024).*
>
> ***Possible mechanistic explanation for the age-related differences in ABRG***
>
> *It is feasible that the age-related differences in ABRG observed in this study stem from the regional effects of beta-adrenergic receptor stimulation (β-ARS), which has been shown to promote heterogeneity in ventricular repolarization (Caldwell et al., 2023). Previous studies have demonstrated that β-ARS substantially increases I_{Ks} activation (Sanguinetti et al., 1991; Volders et al., 2003), and that increased I_{Ks} in the presence of β-ARS leads to more pronounced transmural dispersion of repolarization (Shimizu & Antzelevitch, 1998; Viswanathan et al., 1999). Since I_{Ks} current density is approximately double in apical cardiomyocytes*

compared to basal cardiomyocytes (Szentadrassy et al., 2005), it is plausible that greater β-ARS at faster heart rates leads to larger apicobasal difference in I_{Ks} function and increases the apicobasal dispersion of ventricular repolarization. As aging is associated with a decline in resting heart rate and decreased β-ARS responsiveness (White et al., 1994), the apicobasal difference in I_{Ks} possibly diminishes, leading to gradual disappearance of ABRG. This mechanistic explanation is in line with our findings that show shorter repolarization at the ventricular apex ($RT_{ECGI,apex}$) in younger subjects (Figure 3B) and at faster heart rates (Figure 3F), and the ABRG decline with increasing age (Figure 3A) and at slower heart rates (Figure 3E).

The reviewer is correct that our modeling approach does not take β-ARS into account. This limits the rate dependency of ABRGs in our models. To illustrate the extent of ABRG rate dependency in the ECGI and simulation data, we provide the reviewer with Figure S4. In addition, we state the absence of the β-ARS component in our models as a limitation:

By using ECGI recordings acquired at resting heart rate, our study did not allow for us to separate the effects of age and heart rate on ABRG. For example, the lack of information about the role of β-ARS in our patient cohort prevented us from using a more complex computational approach that would have allowed us to incorporate the impact of β-ARS on the ABRG (Heijman et al., 2011). Thus, future studies should focus on the rate dependency of ABRG, as suggested by our data (Figures 4E), with a particular emphasis on the potential role of β-ARS when patient data are available.

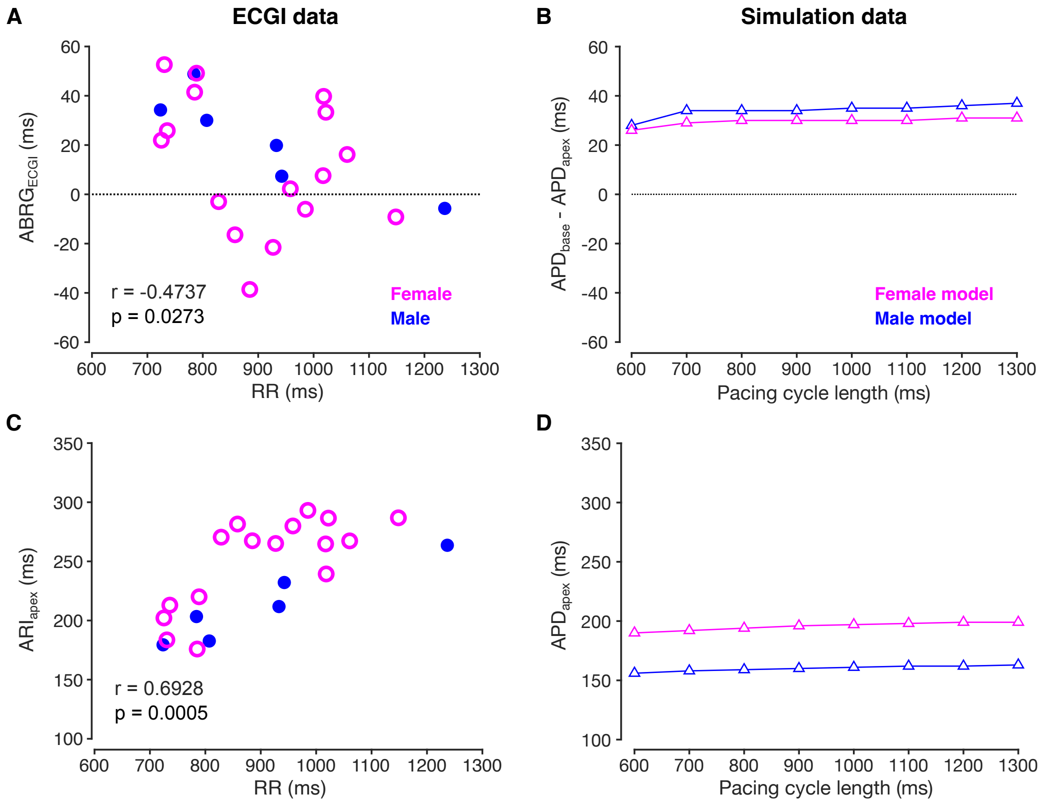

Figure S4. Rate dependency of the apicobasal repolarization gradient (ABRG) in the electrocardiographic imaging (ECGI) and simulation data. (A) ABRG obtained from ECGI data ($ABRG_{ECGI}$), calculated as the difference between activation recovery intervals (ARI) at the base and the apex (ARI_{apex}) was negatively associated with RR interval. The panel is identical to Figure S2E. **(B)** A limited rate dependency was observed for the ABRG, calculated as the action potential duration (APD) difference between the base (APD_{base}) and the apex (APD_{apex}) of the model. **(C)** ARI_{apex} gradually prolonged with RR interval. The panel is identical to Figure S2F. **(D)** A limited rate dependency was observed for APD_{apex} within the same range of pacing rates. The simulations were performed in the male and the female model, using the scaling coefficients of 2.0 for the apex and 1.45 for the base. In both models, the data were obtained during apical pacing.

3. The idea that advanced age could be cardioprotective (regarding ABRG) is very interesting, since it opposes to the natural intuition of ageing and increased cardiovascular risk. I like that the authors linked steep natural ABRGs to idiopathic VF, but could they provide further evidence that idiopathic ventricular arrhythmias are more common in younger individuals to strengthen their finding that steep ABRG might be more responsible for arrhythmias in subjects <50 years of age, compared to older individuals in which other factors (e.g., fibrosis) could play a greater role.

We thank the reviewer for their constructive comment and the great suggestion. After performing a thorough literature search, we found 3 studies on sudden arrhythmic death syndrome (SADS) that report the majority of cases being withing the age range where we observed steep ABRGs (Behr *et al.*, 2007; Mellor *et al.*, 2014; Lahrouchi *et al.*, 2017). This further supports the possible link between ABRGs and lethal ventricular arrhythmias. We rephrased the section dedicated to ABRG as a possible contributor to the ventricular arrhythmogenic substrate in idiopathic VF, and we reference these studies in the manuscript:

ABRG as a possible contributor to the arrhythmogenic substrate in idiopathic VF and sudden cardiac death

The presence of steep natural ABRGs in subjects <50 years of age and the occurrence of arrhythmias in the computational models that incorporated such ABRGs offer a hypothesis that steep natural ABRGs could contribute to the arrhythmogenic substrate in individuals who have experienced idiopathic VF or have suffered sudden cardiac death. Abnormal dispersion of ventricular repolarization has been found in idiopathic VF survivors, and it has been suggested that VF emerges from the interaction of a premature beat and the region with abnormally dispersed repolarization (Cluitmans et al., 2021). Studies on sudden arrhythmic death syndrome (SADS) reported a mean age of 31.4 years (Mellor et al., 2014), 32 years (Behr et al., 2007), and median age of 24 years (Lahrouchi et al., 2017) in the SADS victims. This indicates that the majority of cases occurred within the age range where we observed steep natural ABRGs. Moreover, a gradual decrease in the number of SADS cases can be observed after 40 years of age (Mellor et al., 2014), which corresponds to our finding of ABRGs declining with increasing age, and further supports the potential association between ABRG and SADS. To unravel the possible role of ABRG in SADS and idiopathic VF, studying the interaction of ABRG and Purkinje network would be of particular interest, as Purkinje ectopy has been documented as a frequent arrhythmia trigger in idiopathic VF survivors (Haïssaguerre et al., 2020).

4. I would appreciate if the authors indicated the conduction velocities of the tissue, or the sigma values used to achieve these velocities. The meshes appear to be slightly coarse, with some elements likely exceeding 500 microns of edge length. Thus, stating the velocity values might reassure the reader that those situations of multiple simultaneous re-entrant circuits, among others, are not the result of convergence errors.

We thank the reviewer for this remark. We now state the conduction velocities in the Methods:

Computational models of the human ventricular epicardium

A male and female computational model of the human epicardium were used to simulate the effect of different ABRG levels on arrhythmia vulnerability (Figure 1A). These models were derived from magnetic resonance imaging data of a male and female subject, in line with previously published studies (Moreno et al., 2011; Rivaud et al., 2021). The male model contains 216,910 nodes and 432,926 triangular elements with an average edge length of 383±89 µm. The female model contains 180,060 nodes and 359,269 triangular elements with an average edge length of 393±93 µm. Myocardial fibers (Bayer et al., 2012) and sex-specific ventricular electrophysiology (Figure 1B)(O'Hara et al., 2011; Peirlinck et al., 2021) were incorporated into both models. Tissue conductivity was set according to a previous study (Bayer et al., 2016), resulting in conduction velocities of 89 cm/s along the myocardial fibers and 47 cm/s perpendicular to myocardial fibers at the pacing cycle length of 750 ms.

5. In Line 153, the authors state that they have computed 112 simulations in total. Did the authors mean 112 simulations in each model (224 in total)? There are 56 apicobasal gradients, a male and a female model, and two stimulation locations.

Thank you for pointing this out. Indeed, the text on the line 156 was referring to the number of simulations in each epicardial model. The total number of simulations is 224, as stated by the reviewer. We corrected this information in the manuscript, together with the total number of core-hours required.

> *All simulations were performed as monodomain using the cardiac electrophysiology simulator openCARP (https://opencarp.org) (Plank et al., 2021) running on a DELL workstation (32 cores, Intel Xeon Gold 6226R CPU @ 2.90 GHz, 256 GB of memory) and the Joliot-Curie TGCC supercomputer. Simulation results were visualized with the Meshalyzer software (https://git.opencarp.org/openCARP/meshalyzer). In total, 224 simulations were performed (2 sex-specific meshes, 2 pacing sites, 56 different ABRGs), requiring approximately 14,100 core-hours.*

References

Behr ER, Casey A, Sheppard M, Wright M, Bowker TJ, Davies MJ, McKenna WJ & Wood DA (2007). Sudden arrhythmic death syndrome: A national survey of sudden unexplained cardiac death. *Heart* **93,** 601–605.

Cluitmans MJM et al. (2021). Noninvasive detection of spatiotemporal activation-repolarization interactions that prime idiopathic ventricular fibrillation. *Sci Transl Med* **13,** 9317.

Coronel R, Wilms-Schopman FJG, Opthof T & Janse MJ (2009). Dispersion of repolarization and arrhythmogenesis. *Heart Rhythm* **6,** 537–543.

Lahrouchi N et al. (2017). Utility of Post-Mortem Genetic Testing in Cases of Sudden Arrhythmic Death Syndrome. *J Am Coll Cardiol* **69,** 2134–2145.

Mellor G, Raju H, De Noronha S V., Papadakis M, Sharma S, Behr ER & Sheppard MN (2014). Clinical characteristics and circumstances of death in the sudden arrhythmic death syndrome. *Circ Arrhythm Electrophysiol* **7,** 1078–1083.

Rivaud MR, Bayer JD, Cluitmans M, van der Waal J, Bear LR, Boukens BJ, Belterman C, Gottlieb L, Vaillant F, Abell E, Dubois R, Meijborg VMF & Coronel R (2021). Critical repolarization gradients determine the induction of reentry-based torsades de pointes arrhythmia in models of long QT syndrome. *Heart Rhythm* **18,** 278–287.

Sobota V, Prassl AJ, Ozenne V, Augustin CM, Nordmeyer S & Bayer JD (2022). The interplay of sex-specific electrophysiology and repolarization heterogeneity governs ventricular arrhythmia sustainability in women. In *ESC Congress 2022*, p. 2518. Available at: https://academic.oup.com/eurheartj/article/43/Supplement_2/ehac544.2518/6745744.

Stoks J, Patel KHK, van Rees B, Nguyen UC, Mihl C, Deissler PM, ter Bekke RMA, Peeters R, Vijgen J, Dendale P, Ng FS, Cluitmans MJM & Volders PGA (2024). Variant patterns of electrical activation and recovery in normal human hearts revealed by noninvasive electrocardiographic imaging. *Europace*; DOI: 10.1093/europace/euae172.

Dear Dr Sobota,

Re: JP-RP-2025-288356R1 "The apicobasal dispersion of ventricular repolarization in humans is associated with age and affects arrhythmia vulnerability" by Vladimír Sobota, Job Stoks, Kiran Haresh Kumar Patel, Roshni Shetty, Haibo Ni, Eleonora Grandi, Fu Siong Ng, Paul GA Volders, Matthijs J.M. Cluitmans, and Jason D Bayer

Thank you for submitting your manuscript to The Journal of Physiology. It has been assessed by a Reviewing Editor and by 1 expert referee and we are pleased to tell you that it is acceptable for publication following satisfactory minor revision.

The referee reports are copied at the end of this email.

Please address all the points raised and incorporate all requested revisions. We hope you will find the comments helpful and that you will be able to return your revised manuscript within 4 weeks. If you require longer than this, please contact journal staff: jp@physoc.org.

Your revised manuscript should be submitted online using the link in your Author Tasks Link Not Available. This link is accessible via your account as Corresponding Author; it is not available to your co-authors. If this presents a problem, please contact journal staff (jp@physoc.org). Image files from the previous version are retained on the system. Please ensure you replace or remove any files that are being revised.

If you do not wish to submit a revised version of your manuscript, you must inform our journal staff (jp@physoc.org) or reply to this email to request withdrawal. Please note that a manuscript must be formally withdrawn from the peer review process at one journal before it may be submitted to another journal.

TRANSPARENT PEER REVIEW POLICY: To improve the transparency of its peer review process The Journal of Physiology publishes online, as supporting information, the peer review history of all articles accepted for publication. Readers will have access to decision letters, including Editors' comments and referee reports, for each version of the manuscript, as well as any author responses to peer review comments. Referees can decide whether or not they wish to be named on the peer review history document.

ABSTRACT FIGURES: Authors are expected to use The Journal's premium BioRender account to create/redraw their Abstract Figures. Information on how to access this account is here:
https://physoc.onlinelibrary.wiley.com/journal/14697793/biorender-access.

This will enable Authors to create and download high-resolution figures. If authors have used the free BioRender service, they can use the instructions provided in the link above to download a high-resolution version suitable for publication.
The link provided should only be used for the purposes of this submission. Authors will be charged for figures created on this account if they are not related to this manuscript submission.

LANGUAGE EDITING AND SUPPORT FOR PUBLICATION: If you would like help with English language editing, or other article preparation support, Wiley Editing Services offers expert help, including English Language Editing, as well as translation, manuscript formatting, and figure formatting at www.wileyauthors.com/eeo/preparation. You can also find resources for Preparing Your Article for general guidance about writing and preparing your manuscript at www.wileyauthors.com/eeo/prepresources.

RIGOUR AND REPRODUCIBILITY: We recommend authors consult The Journal's Rigour and Reproducibility requirements to ensure that their manuscript is suitable for publication: https://physoc.onlinelibrary.wiley.com/pb-assets/hub-assets/physoc/documents/TJP-Rigour-and-Reproducibility-Requirements.pdf

REVISION CHECKLIST:
Check that your Methods section conforms to journal policy: https://jp.msubmit.net/cgi-bin/main.plex?form_type=display_requirements#methods

Check that data presented conforms to the statistics policy: https://jp.msubmit.net/cgi-bin/main.plex?form_type=display_requirements#statistics

Upload a full Response to Referees file. To create your 'Response to Referees' copy all the reports, including any comments from the Senior and Reviewing Editors, into a Microsoft Word, or similar, file and respond to each point, using font or background colour to distinguish comments and responses and upload as the required file type.

Please upload two versions of your manuscript text: one with all relevant changes highlighted and one clean version with no changes tracked. The manuscript file should include all tables and figure legends, but each figure/graph should be uploaded as separate, high-resolution files. The journal is now integrated with Wiley's Image Checking service. For further details, see: https://www.wiley.com/en-us/network/publishing/research-publishing/trending-stories/upholding-image-integrity-wileys-image-screening-service

You may also upload:
- 'Potential Cover Art' for consideration as the issue's cover image
- Appropriate Supporting Information (Video, audio or data set: see https://jp.msubmit.net/cgi-bin/main.plex?form_type=display_requirements#supp).

We look forward to receiving your revised submission.

If you have any queries, please reply to this email and we will be pleased to advise.

Yours sincerely,

Bjorn Knollmann
Senior Editor
The Journal of Physiology

REQUIRED ITEMS

- You must start the Methods section with a paragraph headed Ethical Approval. If experiments were conducted on humans, confirmation that informed consent was obtained, preferably in writing, that the studies conformed to the standards set by the latest revision of the Declaration of Helsinki and that the procedures were approved by a properly constituted ethics committee, which should be named, must be included in the article file. If the research study was registered (clause 35 of the Declaration of Helsinki), the registration database should be indicated, otherwise the lack of registration should be noted as an exception (e.g. 'The study conformed to the standards set by the Declaration of Helsinki, except for registration in a database'). For further information see: https://physoc.onlinelibrary.wiley.com/hub/human-experiments.

EDITOR COMMENTS

Reviewing Editor:

I agree that the comments were addressed satisfactorily in the manuscript.

Senior Editor:

This manuscript is acceptable once compliance with clause 35 is stated. Please see 'Required Items' above.

REFEREE COMMENTS

Referee #1:

The authors have appropriately addressed all my comments.

END OF COMMENTS

Response to Reviewers

We thank the reviewer and editors for their positive feedback. For clarity, we have colored the reviewer's comments in blue, added/edited text in the manuscript in red, original manuscript text in gray, and the text of the rebuttal in black.

Required Items

You must start the Methods section with a paragraph headed Ethical Approval. If experiments were conducted on humans, confirmation that informed consent was obtained, preferably in writing, that the studies conformed to the standards set by the latest revision of the Declaration of Helsinki and that the procedures were approved by a properly constituted ethics committee, which should be named, must be included in the article file. If the research study was registered (clause 35 of the Declaration of Helsinki), the registration database should be indicated, otherwise the lack of registration should be noted as an exception (e.g., 'The study conformed to the standards set by the Declaration of Helsinki, except for registration in a database').

The first paragraph of the Methods section has been modified to comply with the requirements. The clinical trial numbers are now provided for both ethics committee approvals:

> *This study is based on previously recorded ECGI data (Stoks et al., 2024). It was approved by the local ethics committees of Maastricht University Medical Center + (MUMC+), The Netherlands (METC 11-2-043, registered at ClinicalTrials.gov, identifier number NCT03947021), and the Health Research Authority London (Surrey) Research Ethics Committee, United Kingdom (19/LO/0762, registered at ClinicalTrials.gov, identifier number NCT03910725). The study adheres with the Declaration of Helsinki. Written informed consent was obtained from all subjects prior to inclusion.*

Editor Comments

Reviewing Editor: I agree that the comments were addressed satisfactorily in the manuscript.
We thank the Reviewing Editor for their positive comment and the time they invested in reviewing our manuscript.

Senior Editor: This manuscript is acceptable once compliance with clause 35 is stated. Please see 'Required Items' above.
We thank the Senior Editor for their positive feedback and the time invested in reviewing our manuscript. We have rephrased the first paragraph of the Methods section to comply with clause 35. The detailed description of the edits is provided above.

Referee Comments

The authors have appropriately addressed all my comments.
We thank the Referee again for their valuable feedback. We appreciate the time they invested in reviewing our manuscript.

Dear Dr Sobota,

Re: JP-RP-2025-288356R2 "The apicobasal dispersion of ventricular repolarization in humans is associated with age and affects arrhythmia vulnerability" by Vladimír Sobota, Job Stoks, Kiran Haresh Kumar Patel, Roshni Shetty, Haibo Ni, Eleonora Grandi, Fu Siong Ng, Paul GA Volders, Matthijs J.M. Cluitmans, and Jason D Bayer

We are pleased to tell you that your paper has been accepted for publication in The Journal of Physiology.

The last Word (or similar) version of the manuscript provided will be used by the Production Editor to prepare your proof. When this is ready you will receive an email containing a link to Wiley's Online Proofing System. The proof should be thoroughly checked and corrected as promptly as possible.

Authors should note that it is too late at this point to offer corrections prior to proofreading. Major corrections at proof stage, such as changes to figures, will be referred to the Editors for approval before they can be incorporated. Only minor changes, such as to style and consistency, should be made at proof stage. Changes that need to be made after proof stage will usually require a formal correction notice.

All queries at proof stage should be sent to: TJP@wiley.com.

Following copyediting and typesetting the accepted version will be published online.

Yours sincerely,

Bjorn Knollmann
Senior Editor
The Journal of Physiology

IMPORTANT POINTS TO NOTE FOLLOWING YOUR PAPER'S ACCEPTANCE:

If you would like to receive our 'Research Roundup', a monthly newsletter highlighting the cutting-edge research published in The Physiological Society's family of journals (The Journal of Physiology, Experimental Physiology, Physiological Reports, The Journal of Nutritional Physiology and The Journal of Precision Medicine: Health and Disease), please click this link, fill in your name and email address and select 'Research Roundup':
https://www.physoc.org/journals-and-media/membernews

• TRANSPARENT PEER REVIEW POLICY: To improve the transparency of its peer review process, The Journal of Physiology publishes online as supporting information the peer review history of all articles accepted for publication. Readers will have access to decision letters, including Editors' comments and referee reports, for each version of the manuscript as well as any author responses to peer review comments. Referees can decide whether or not they wish to be named on the peer review history document.

• You can help your research get the attention it deserves! Check out Wiley's free Promotion Guide for best-practice recommendations for promoting your work at: www.wileyauthors.com/eeo/guide. You can learn more about Wiley Editing Services which offers professional video, design, and writing services to create shareable video abstracts, infographics, conference posters, lay summaries, and research news stories for your research at: www.wileyauthors.com/eeo/promotion.

• IMPORTANT NOTICE ABOUT OPEN ACCESS: To assist authors whose funding agencies mandate public access to published research findings sooner than 12 months after publication, The Journal of Physiology allows authors to pay an Open Access (OA) fee to have their papers made freely available immediately on publication.

The Corresponding Author will receive an email from Wiley with details on how to register or log-in to Wiley Authors Services where you will be able to place an order.

• You can check if your funder or institution has a Wiley Open Access Account here: https://authorservices.wiley.com/author-resources/Journal-Authors/licensing-and-open-access/open-access/author-compliance-tool.html.

EDITOR COMMENTS

The manuscript is now acceptable. Thank you for your excellent contribution!
